# Novel *Trichoderma* Isolates Alleviate Water Deficit Stress in Susceptible Tomato Genotypes

**DOI:** 10.3389/fpls.2022.869090

**Published:** 2022-05-02

**Authors:** Ranjana Rawal, Joseph C. Scheerens, Sean M. Fenstemaker, David M. Francis, Sally A. Miller, Maria-Soledad Benitez

**Affiliations:** ^1^Department of Horticulture and Crop Science, Ohio Agricultural Research and Development Center, The Ohio State University, Wooster, OH, United States; ^2^Department of Plant Pathology, Ohio Agricultural Research and Development Center, The Ohio State University, Wooster, OH, United States

**Keywords:** abiotic stress, fungi, plant growth-promoting microbes, PEG, arid region, horticulture

## Abstract

Symbiotic fungi in the genus *Trichoderma* can induce abiotic stress tolerance in crops. The beneficial effects of *Trichoderma* on water deficit stress are poorly understood and may be isolate-specific. Our objective was to evaluate a collection of Nepalese *Trichoderma* isolates and their efficacy to improve tomato (*Solanum lycopersicum*) growth under water deficit. Variable growth in low moisture environments was observed among *Trichoderma* isolates from Nepal, Ohio, and commercial sources using *in vitro* assays. The overall performance of the population decreased when cultured under conditions of decreasing matric water potential (0.0, –2.8, –4.8, and –8.5 Ψ). Twelve isolates were selected for evaluation for their potential to elicit drought tolerance in greenhouse-grown ‘Roma Organic’ tomatoes. Plants treated with *T. asperelloides*-NT33 had higher shoot weight than the non-inoculated control (T0) under water deficit stress conditions. Further, the stress-reducing efficacy of isolates *T. asperelloides*-NT33, *T. asperellum-*NT16, *T. asperelloides*-NT3, and commercial *T. harzianum-*T22 were tested on tomato genotypes with differing tolerance to drought [‘Roma Organic,’ ‘Jaune Flamme,’ and ‘Punta Banda’]. The water deficit susceptible genotypes ‘Roma Organic’ and ‘Jaune Flamme’ inoculated with isolate NT33 had significantly higher shoot weight (37 and 30% respectively; *p* < 0.05) compared to the non-inoculated control under water deficit stress conditions. In drought tolerant ‘Punta Banda,’ shoot weight was also significantly greater in NT33 inoculated plants under water deficit stress conditions, but with lower magnitude difference (8%; *p* < 0.05). Our results demonstrate differences in the ability of *Trichoderma* isolates to confer tolerance to water deficit in tomato with NT33 potentially relieving stress. Tomato genotypes also play a role in the outcome of interactions with the *Trichoderma* isolates we tested.

## Introduction

By 2050, water consumption for agricultural practices will increase by 60% to support a growing world population (Boretti and Rosa, 2019). Concurrently, drought is becoming a dominant environmental stressor for crop production globally, limiting food security with precarious economic and sociological impacts (Trenberth et al., 2014). Due to climate change, water deficit is spreading to regions where drought was infrequent in the past (Somerville and Briscoe, 2001). Globally, the reduction in yield due to drought is likely to exceed the combined loss of all other possible causes of yield decline (Blum, 1996; Foolad, 2007). Therefore, there is a need to identify solutions to mitigate water deficit stress and its impact on food security.

Tomato (*Solanum lycopersicum* L.) is the world’s second most important vegetable crop in per capita consumption (Singh et al., 2018). On a global scale, tomatoes are grown on 5 million hectares of land with an annual production of about 180 million tons, and a production value of more than 90 billion USD (FAO, 2022). Currently, drought is a significant environmental stressor challenging tomato productivity in areas of inadequate or erratic rainfall that lack irrigation options (Petrozza et al., 2014; Pal et al., 2016). Drought decreases crop production on 25% of arable land throughout the world. Thus, where tomatoes are produced under rainfed agricultural conditions, there is a need to identify multi-faceted, low-cost, environmentally friendly, and sustainable solutions to water deficit that can be implemented on a short-term basis. Symbiotic associations with stress-mitigating soil microorganisms may serve as one of those solutions.

Naturally occurring beneficial soil microorganisms, including members of the genus *Trichoderma* (Family – Hypocreaceae), are studied as accessible and sustainable tools for enhancing plant growth and mitigating biotic and abiotic stressors in both research and commercial production settings (Van Wees et al., 2008; Grover et al., 2011; Kim et al., 2012; Meena et al., 2017; Khadka and Miller, 2021). As such, specific commercial *Trichoderma* isolates are widely used as effective biocontrol agents, biofertilizers, and phyto-stimulators (Harman, 2011; Hermosa et al., 2012; Waghunde et al., 2016; Kashyap et al., 2017; Khan et al., 2017), but evidence of contributions to abiotic stress amelioration in crop species is sparse. Several recent studies suggest that *Trichoderma* inoculation of water-stressed plants enhanced growth by improving root biomass, enhancing water holding capacity, and mobilizing nutrients (Mastouri et al., 2010; Harman, 2011; Bakhshandeh et al., 2020). Further, *Trichoderma* inoculated plants exhibit delayed wilting, increased stomatal conductance, enhanced leaf chlorophyll content, and greater net photosynthesis levels under water deficit stress conditions (Bae et al., 2009; Shukla et al., 2012; Alwhibi et al., 2017; Harman et al., 2019).

Plant genotype influences the degree of colonization and crop stress-mitigating potential of *Trichoderma* even for the established commercial isolates (Harman et al., 2004; Prashar and Vandenberg, 2017). The beneficial effects of *Trichoderma*, however, are more pronounced when plants are under stress compared to when grown under optimal conditions (Mastouri et al., 2010; Lombardi et al., 2018). As it has been demonstrated for other fungi, there is also potential that changes in climate patterns can influence *Trichoderma* distribution and abundance, as well as the outcome of the plant–microbial interaction (Compant et al., 2010; Tyagi et al., 2014; Delgado-Baquerizo et al., 2020). However, whether different species or isolates of *Trichoderma* have adapted as symbionts in stress environments remains unclear.

The overall goal of this research was to evaluate the ability of novel isolates from several species of *Trichoderma*, primarily native to different agroecological regions in Nepal, to improve growth in different tomato genotypes exposed to water deficit stress. Our study involved a series of experiments focused on: (1) assessing these novel isolates for their desiccation tolerance and their efficacy for alleviating water deficit stress symptoms (e.g., reduced biomass accumulation and diminished physiological function) in tomato genotypes differing in water deficit tolerance; (2) delineating the physiological (e.g., relative water content, electrolyte leakage, and photosynthetic efficiency) and biochemical (e.g., total and individual phenolic, proline and hormonal level) changes in leaves of a *Trichoderma*-inoculated, susceptible tomato genotype subjected to water stress; and (3) identifying and mapping genes differentially expressed in leaves of the inoculated tomato genotype under water stress and irrigated conditions (Rawal, 2021). Herein, we report our assessment of the novel *Trichoderma* isolates as plant growth promoters with the potential to alleviate growth-related and physiological stress symptoms across tomato genotypes that differ in water deficit stress tolerance. We hypothesized that (a) *Trichoderma* isolates will differ in their efficacy to diminish the effects of water deficit stress on tomato plant growth; (b) that isolates collected from the drier regions will tolerate desiccation conditions and have a greater impact on water deficit stress amelioration than those from wetter regions, and (c) that the effect of selected *Trichoderma* isolates on growth and water deficit stress will depend on tomato genotype. To ascertain the validity of the latter, we pursued a supporting goal of surveying heirloom and land races within The Ohio State University tomato germplasm collection for water deficit susceptibility or tolerance with the aim of including one or more of these genotypes in our *Trichoderma* trials.

## Materials and Methods

**FIGURE 1:**
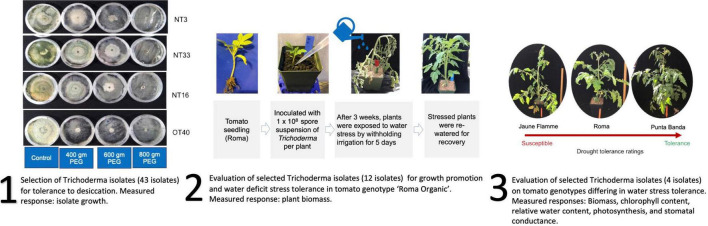
Overview of the replicated experiments reported in this manuscript. First, a collection of isolates from different geographical regions in Nepal were screened for desiccation tolerance. Second, a subset of these isolates was selected to screen for water deficit amelioration in ‘Roma Organic’ tomato. And third, the superior isolates were tested to evaluate *Trichoderma* × tomato genotype interaction in water deficit stress amelioration. Response variables measured in each set of experiments is included in the figure.

Nepalese *Trichoderma* isolates used in this study were imported under the US Department of Agriculture Animal and Plant Health Inspection Service (APHIS) permit #P526P-19-03121. All experimental procedures involving fungal isolates were approved by The Ohio State University Institutional Biosafety Committee as described in Protocol 2019R00000043. These experiments were performed in BSL-2 rated laboratories and greenhouses. A general overview of the experiments reported in this work is shown in Figure 1.

### Selection of *Trichoderma* Isolates for Tolerance to Desiccation

Forty-one different *Trichoderma* isolates collected from diverse agroecological regions of Nepal (designated as NT1-NT39, NT42, NT43) and two *Trichoderma* isolates from Ohio (OT41, OT42) were selected for this study. Molecular and morphological characterization of all isolates used in this study is presented in Khadka et al. (2022). Nepalese *Trichoderma* isolates were recovered from diverse agricultural soils found at environmentally diverse sites (arid, saline, alpine regions), representing potential reservoirs of microorganisms with traits that may benefit plant hosts when grown under differing environmental conditions (Supplementary Figure 1 and Supplementary Table 1). All isolates were evaluated for their capacity to tolerate and grow under severe desiccation conditions. A commercial isolate, *T. harzianum* Rifai (T22) was used as a positive control. This isolate can induce systemic resistance in plants and is available commercially as a bio-stimulant (Mastouri et al., 2010; Harman, 2011). These *Trichoderma* isolates were maintained and preserved on silica gel (Samuels, 2015), and isolates were recovered on Potato Dextrose Agar (PDA) plates (Sigma- Aldrich Inc., St. Louis, MO, United States) at 25°C with a 12 h light/dark cycle for seven days.

Desiccation conditions were created by dissolving polyethylene glycol 8000 MW (PEG) in Potato Dextrose Agar (PDA) medium (Sigma- Aldrich Inc., St. Louis, MO, United States) according to the procedure described by Aujla and Paulitz (2017). Different matric water potentials (0.0, –2.8, –4.8, and –8.5 Ψ) were generated by adding 0, 400, 600, and 800 g PEG (8,000 MW, Fisher Scientific Co., Pittsburgh, PA, United States) into 1 l of PDA, respectively. Each medium’s water potential was determined by using a WP4C dew point potentiometer (Decagon Devices, Inc., Pullman, WA, United States). PDA medium without PEG served as a non-desiccation control. Sterilized PDA + PEG mixtures in 10 ml quantities were poured into individual sterile Petri dishes. A 10 cm diameter sterile cellophane disk (Bio-Rad Laboratories Inc., San Francisco, CA, United States) was embedded into each treatment and control Petri dish containing semi-solid PDA + PEG to provide a solid support for the growth of each *Trichoderma* isolate (Ruano Rosa and López Herrera, 2009). For the matric potential treatments, plates were inoculated with a 5 mm diameter PDA disk of mycelia of an actively growing *Trichoderma* culture. The Petri dishes were sealed with Parafilm and were then incubated at 28°C for seven days. The growth of the *Trichoderma* colony was measured (cm) along two diameters perpendicular to each other (D1 and D2) and the average of these two values was calculated (D1 + D2/2). A total of 44 isolates were evaluated for desiccation tolerance. For each isolate, the desiccation treatment was replicated three times, and plates were arranged in a completely randomized design. The isolates were ranked at each osmotic stress condition based on colony diameter. Ranked colony diameters were correlated among treatment groups in individual responses to increased osmotic stress. The experiment was performed twice.

### Selection of Water Deficit Susceptible Tomato Genotypes

We tested 70 heirloom and landrace tomato (*Solanum lycopersicum* L.) varieties from the germplasm collection of The Ohio State University Tomato Genetics and Breeding Program for their susceptibility or tolerance to water deficit stress conditions. Plants with 3-5 expanded leaves were transferred to 3.7-l plastic containers filled with PRO-MIX (Premier Horticulture, Quakerstown, PA, United States), and spaced 30 cm apart on a greenhouse bench. Growing conditions were as follows: 27°C Day/25°C night; 16/8-h day/night photoperiod; 260 μmol average daily light integral (DLI); 51% average relative humidity; and bi-weekly fertigation with 20N-20P_2_O_5_-20K_2_O (J.R. Peters Inc., Allentown, PA, United States) at a concentration of 1,000 mgL^–1^ twice per week. When the plants reached the growth stage of 6–8 expanded leaves, irrigation was withheld, and plants were evaluated for 6 days. The leaf turgor status of individuals under water deficit stress conditions was assessed daily on a whole plant basis. Pots were weighed at saturation and over the course of the germplasm screen (72 and 144 h after irrigation was halted). Turgor ratings ranged from 1 to 5 (5 = turgid, 4 = soft to the touch, 3 = beginning to wilt, 2 = wilted with complete loss of turgor, and 1 = dead) consistent with previous studies (Waterland et al., 2010). Leaf surface temperatures (°C) of two fully expanded leaves per plant were monitored daily using an infrared thermometer (Zhuhai JiDa Huapu Instrument Hong Kong, SAR China). The experiment was conducted as an augmented design (Federer and Raghavarao, 1975) with two replicates of each variety and 30 replicates of processing tomato variety OH824 (Berry et al., 1991) as an over-replicated, relatively moisture stress-tolerant control.

### Evaluation of Selected *Trichoderma* Isolates for Growth Promotion and Water Deficit Stress Tolerance in Tomato Genotype ‘Roma Organic’

#### Plant Material

The tomato genotype ‘Roma Organic’ (W. Atlee Burpee & Co, Warminster, PA, United States), reported as drought susceptible and able to support *Trichoderma* colonization (Vinale et al., 2013; Aghaie et al., 2018), was selected for this experiment. Tomato seeds were surface sterilized by dipping in 10% sodium hypochlorite, followed by 95% ethanol for five minutes. Seeds were then rinsed (5 X) successively in sterile distilled water. The seeds were sown in 98-cell plastic plug trays containing an in-house soil mix. Growing substrate for seed germination, and water deficit stress experiments was prepared using a mix of composted sandy soil and peat (Fafard Sphagnum Peat Moss, Sun Gro Horticulture Products, Agawam, MA, United States) (6:1) plus 1.8 g liter^–1^ lime and 0.45 g liter^–1^ fertilizer 20N-20P_2_O_5_-20K_2_O (Jack’s professional All- Purpose Fertilizer, JR Peters INC., Allentown, PA, United States), steamed sterilized for 12 h at 18 psi and 121°C. The growing substrate tested negative for the presence of *Trichoderma* through dilution plating on *Trichoderma* selective media. For germination and initial seedling growth, trays were kept in a greenhouse at 25°C with 16/8-h day/night photoperiod and 65% relative humidity for two weeks.

#### Inoculum Preparation of Fungal Isolates and Treatment Designation

**FIGURE 2:**
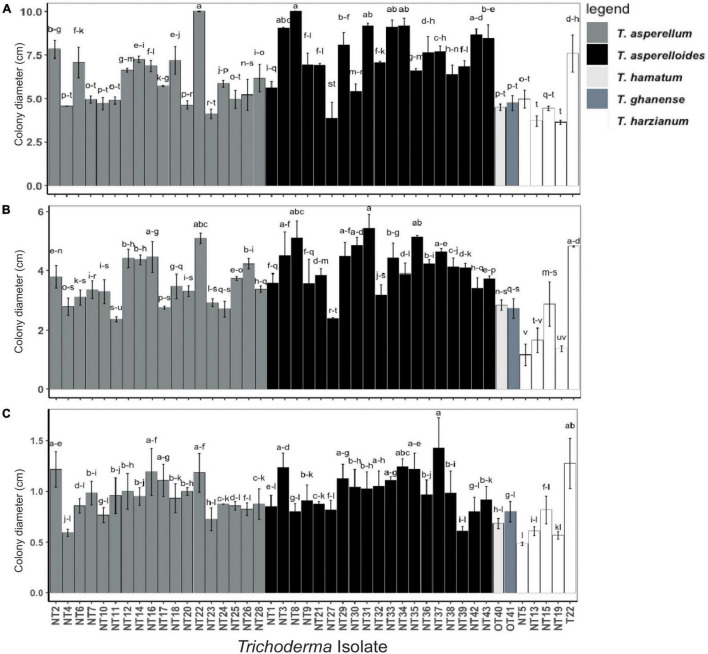
Radial mycelial growth of *Trichoderma* isolates grown in media at different matric water potentials: **(A)** –2.8 Ψ, **(B)** –4.8 Ψ, and **(C)** –8.5 Ψ; the matric potentials were generated by adding 400, 600, and 800-g PEG into 1-l PDA respectively. Radial growth of the *Trichoderma* colony (cm) was measured along the plate diameter after 7 days. Values represent the mean growth ± SE of isolates from two trials each performed in triplicate. Isolates with different superscripts are significantly different according to Fisher LSD test (*P* < 0.05). Bar colors represent the species of tested isolates.

Eleven novel isolates of *Trichoderma* (NT3, NT14, NT16, NT21, NT22, NT31, NT33, NT37, OT40, OT41, NT43), belonging to *T. asperellum*, *T. asperelloides*, *T. hamatum*, or *T. ghanense*, (Figure 2) were selected for a greenhouse screening based on differing ability to proliferate under reduced matric water potentials (i.e., low- and high-performing isolates) and to represent environmental diversity in isolate origin. These 11 isolates and the commercial *Trichoderma* T22 (Positive control) were cultured on Potato Dextrose Agar (PDA) medium (Sigma- Aldrich Inc., St. Louis, MO, United States) at 25°C for 14 days with 12 h light/dark cycle. Conidia were recovered by flooding each plate with sterile distilled water and gently scraping the colony surface with a sterile toothpick. The *Trichoderma* suspensions were collected on a sterile 15 ml tube and vortexed briefly. The hyphae and mycelia were removed by passing the suspensions through four layers of sterilized cheesecloth (Al-Hazmi and TariqJaveed, 2016). The concentration of *Trichoderma* conidia was determined using a hemocytometer. The suspension was adjusted to 1 × 10^8^ conidia per ml by adding sterile distilled water.

Seedlings were transplanted into 14 cm plastic pots containing steam-sterilized soil substrate. The plants were inoculated with *Trichoderma* isolates twice during the experiment. First, at seedling transplanting, plants were inoculated by soaking the roots of each 2-week-old seedling in the conidia suspension (1 × 10^8^ conidia per plant) for 1 h prior to transplant, according to Alwhibi et al. (2017). A second *Trichoderma* inoculation was performed two weeks after transplanting, with a 1 ml suspension of 1 × 10^8^ conidia applied with a pipette to the soil, close to the base of the plant. Non-inoculated control plants (T_0_) were treated similarly, but with sterile distilled water. The greenhouse conditions were set as follows: 27°C Day/25°C night; 16/8-h day/night photoperiod; 260 μmol average daily light integral (DLI); and 65% average relative humidity. The seedlings were fertilized with 20N-20P_2_O_5_- 20K_2_O (Jack’s professional All- Purpose Fertilizer, JR Peters INC., Allentown, PA, United States) at a concentration of 2 g liter^–1^ fertilizer once per week.

In the greenhouse the experiment was arranged as a randomized factorial block design with two treatments. The treatment levels were as follows:

Irrigation treatment — continuously irrigated or subjected to water deficit stress.

*Trichoderma* inoculation — T_0_, no inoculation, NT3, NT14, NT16, NT21, NT22, NT31, NT33, NT37, OT40, OT41, NT43, T22.

The 26 treatment combinations (i.e., irrigation regimen X *Trichoderma* inoculation status) were represented by a single plant (and one plant per pot) in each of six blocks for a total of 156 plants. The experiment was repeated two times.

#### Induction of Water Deficit Stress

All plants were irrigated daily with 500 ml of water per pot prior to inducing water deficit stress. Three weeks after transplanting, replicates representing each inoculation treatment (i.e., inoculated and control plants) were randomly assigned to irrigation treatment groups with six replicates subjected to artificial water deficit stress and six irrigated daily, serving as water deficit stress controls. Water deficit stress was created by withholding irrigation for 5 days. After 5 days, the plants were again irrigated with 500 ml of water in each pot once a day for the next 7 days for recovery.

The soil water potential was measured and recorded through the duration of the experiment (transplanting to sampling) using the Soil Moisture Smart Sensor (S- SMx-M005, Onset Computer Corporation, Bourne, MA, United States) and the HOBO USB Micro Station Data Logger (Onset Computer Corporation, Bourne, MA, United States). Twelve moisture sensors were inserted in the pots with four different treatments (non-inoculated control + water-stressed, commercial *Trichoderma*-T22 + water-stressed, non-inoculated control + irrigated, commercial *Trichoderma*-T22 + irrigated) in three replications and the water content in the pots was recorded before water deficit stress, during water deficit stress, and after recovery. After one week of re-watering and recovery, individual plants from both irrigation treatment groups were harvested for shoot and root weight measurements. For this, shoots were cut at the base of growth, and the root balls were excavated from the pots and intact and broken roots were carefully collected. The roots were cleaned thoroughly to remove any attached growing substrate prior to weighing.

### Evaluation of Selected *Trichoderma* Isolates on Tomato Genotypes Differing in Water Stress Tolerance

#### Plant Material and Treatment Designation

Tomato varieties ‘Punta Banda’ (water deficit tolerant; Native Seeds/SEARCH, Tucson, AZ, United States), ‘Roma Organic’ (water deficit susceptible), and ‘Jaune Flamme’ (water deficit susceptible, OSU Tomato Genetics and Breeding Program) were selected for this experiment. ‘Punta Banda’ and ‘Roma Organic’ were chosen based on tolerance ratings reported by Aghaie et al. (2018) and the water deficit susceptibility of ‘Jaune Flamme’ was determined as part of this research.

The three Nepalese *Trichoderma* isolates (NT3, NT16, NT33) and the T22 (positive control) were selected based on variability in performance during the greenhouse screening of *Trichoderma* isolates in ‘Roma Organic’ tomato genotype (see section “Evaluation of Selected *Trichoderma* Isolates for Growth Promotion and Water Deficit Stress Tolerance in Tomato Genotype ‘Roma Organic”’). This experiment was conducted using a randomized factorial block design with three treatment factors and nine replications per treatment combination.

The treatments and levels were as follows:

Irrigation treatment — continuously irrigated or subjected to water deficit stress.

*Trichoderma* inoculation — no inoculation (T_0_), NT3, NT16, NT33, T22.

Tomato genotype — ‘Jaune Flamme,’ ‘Roma Organic,’ ‘Punta Banda.’

The 30 treatment combinations (i.e., irrigation regimen X *Trichoderma* inoculation status X three genotypes) were represented by a single plant (one plant per pot) in each of the nine blocks for a total of 270 plants. The experiment was repeated two times.

*Trichoderma* inoculation procedures, assessment of plants to irrigation treatment groups and water deficit regimens followed those described under the experiment 1. After one week of re-watering and recovery, all plants from all treatment groups were harvested for the following growth measurements: total number of leaves per plant, shoot fresh weight, and root fresh weight.

#### Plant Physiological Analysis

Plant physiological parameters including chlorophyll content, relative water content, photosynthesis, and stomatal conductance were measured in leaves at different stages of the experiment. Chlorophyll content in leaves was estimated with a Soil Plant Analysis Development meter (SPAD 502 Plus, Konica Minolta, Inc., Ramsey, NJ, United States). The SPAD meter records the absorbance at near red light (650 nm) and at infrared (940 nm) regions of the leaves. Based on these two absorbance, the instrument calculates a numerical soil plant analysis development (SPAD) value which is proportional to the chlorophyll content in the leaf (Uddling et al., 2007). SPAD readings were recorded between 9.00 and 11.00 a.m. from the upper three leaves after two days of recovery. The average of the three leaves per plant was used in the analysis.

To measure the leaf relative water content (RWC), a cork borer was used to remove five 5 mm disks from the upper third leaf on the 5th day of water deficit stress. The fresh weight (FW) of the sampled disks was recorded. Afterward, the disks were soaked in distilled water for 4 h at room temperature to achieve maximum turgidity and turgid weight was recorded (TW). Finally, the dry weight (DW) of the disks was recorded after 24 h of oven-drying at 80°C. RWC was calculated by using the following formula: RWC (%) = (FW-DW)/(TW-DW) × 100.

Photosynthesis and stomatal conductance were measured to evaluate the physiological health of water deficit stressed plants and influence of *Trichoderma* inoculation. The portable photosynthetic system Li- 6400X (LI-COR Inc, Lincoln, NE, United States) was used to measure these physiological parameters. For this, the fully expanded third leaf from the top of a tomato plant was selected and the measurements were recorded between 9.00 am and 1:00 p.m. at the 4th day of water deficit stress. For physiological parameters, the measurements were taken from three replications per treatment combination.

### Statistical Analysis

The difference between the treatments were evaluated using the linear mix model function “lmer” using the package lme4 (Bates et al., 2014) in R (R Core Team, 2013; Mendiburu, 2015), where experimental run was treated as a random factor in *in vitro* experiment. The normality of the data was assessed using the Shapiro–Wilk test followed by the Bartlett test to check the homogeneity of variance using the functions shapiro.test and bartlett.test in R (Kassambara, 2020). Spearman (rank) correlations were used to compare the growth of isolates at different matric water potentials and these relationships were graphed using the *ggpubr* package (0.4.0) (Kassambara, 2020). To evaluate water deficit tolerance of tomato varieties, best linear unbiased predictors (BLUPs) were extracted from heirloom and landrace tomato raw water deficit stress data using the package lme4 (Bates et al., 2014) to adjust mean values according to greenhouse spatial differences observed in the performance of the replicated control. BLUP-adjusted means were used to create a vector for maximum leaf temperature and maximum wilt phenotypes. A linear mixed model was fitted to the data to determine the effects of *Trichoderma* isolate and irrigation main effects and their interactions on the response variables in greenhouse screening in tomato genotype ‘Roma Organic.’ Similarly, a linear mixed model was fitted to the data to determine the main effects and their interactions for *Trichoderma* isolates, tomato genotypes, and irrigation regimens on the response variables in the last greenhouse experiment. Experimental run was considered a random-effect factor and other factors were considered fixed effects. Type III *F* tests were used for determining significance of effects. Interaction means were partitioned with slices of means to determine the effects of isolate and genotype at each of the irrigation treatments (Schabenberger et al., 2000; Stroup et al., 2018). With slices, an overall *F* test was performed for each irrigation treatment, and means were compared in a pairwise fashion at each level of irrigation treatment. The GLIMMIX procedure of SAS was used for the analysis.

## Results

### Desiccation Tolerance of *Trichoderma* Isolates

In the *in vitro* screening procedure, all *Trichoderma* isolates tested on PDA medium without PEG (negative control, 0.0Ψ) were viable and covered the 10 cm Petri plate’s surface within 7 days after inoculation. However, the colony diameter (mycelial growth) of all *Trichoderma* isolates tested decreased with increasing negative matric water potential as evidenced by the means and standard deviations of the isolate population cultured at –2.8 Ψ (6.47 ± 1.78 cm, Figure 2A), –4.8 Ψ (3.63 ± 1.01 cm, Figure 2B), and –8.5 Ψ (0.93 ± 0.21 cm, Figure 2C). Individuals within the population of isolates exhibited continuous variability in their ability to proliferate at each osmotic stress-imposed level. Ranked isolate colony diameters were significantly correlated among treatment groups [rho (ρ) = 0.72 and 0.60 for treatments –2.8 vs. –4.8 and vs. –8.5 Ψ, respectively and rho (ρ) = 0.64 for treatments –4.8 vs. –8.5 Ψ] indicating a degree of homogeneity in individual responses to increased osmotic stress (Supplementary Figures 2A–C). Isolates NT3, NT16, NT22, NT29, NT31, NT33, NT37, and T22 exhibited colony diameters that ranked within the upper one-third (i.e., rank performance > 15) of the population at each level of osmotic stress. Similarly, Nepalese isolates NT4, NT5, NT10, NT13, NT15, NT19, NT23, and NT27 and the Ohio isolates OT40 and OT41 grew less under all levels of osmotic stress, ranking within the lower one-third (i.e., rank performance ≤ 30) of the population at each level. Different patterns of growth under osmotic stress were observed. For instance, NT8 ranked 2nd and 3rd among the isolates for its ability to proliferate at –2.8 and –4.8 Ψ, respectively. However, its growth appeared to be constrained at the highest level of osmotic stress (i.e., 8.5 Ψ), suggesting limits to its adaptive response. Conversely, NT16 grew modestly at –2.8 Ψ but it successively gained in rank performance compared to other isolates when challenged at –4.8 and –8.5 Ψ. Individual isolate growth (value ± standard error and rank) measurements at each osmotic stress level are displayed in Supplementary Table 2.

### Water Deficit Tolerance/Susceptibility Among Tomato Heirloom Varieties and Landraces

Continuous variation in BLUP-adjusted mean leaf temperatures was observed among the 70 genotypes subjected to 6 days of water deficit stress (Supplementary Figure 3A) ranging from –0.9 (water deficit stress-tolerant) to 0.6 (water deficit stress-susceptible). The ability to maintain leaf turgor pressure during water deficit also differed among genotypes with BLUP-adjusted means ranging from –1.0 (water deficit stress-susceptible) to 0.9 (water deficit stress-tolerant) (Supplementary Figure 3B). No significant differences in substrate water loss were detected due to genetic effects (genotype) or environmental effects (row and column). Leaf temperature and turgor maintenance data were complementary [rho (ρ) = –0.73]. OH8245 ranked 9th in ability to maintain leaf temperature and 14th in the ability to maintain leaf turgor confirming its designation as moderately water deficit stress tolerant.

Among the water deficit stress-susceptible genotypes, the heirloom variety ‘Jaune Flamme’ was selected for inclusion in experiments examining differences in tomato cultivar response to *Trichoderma* inoculation described below. This orange-fruited variety is used regionally by farm market growers, has been studied as a source of β-carotene (provitamin A) in fruit (Orchard et al., 2021) and we were familiar with its horticultural properties. In the water deficit tolerance/susceptibility screen reported here, ‘Jaune Flamme’ ranked 57th in its ability to maintain leaf temperature and 60th in its ability to maintain leaf turgor under water deficit out of 70 genotypes tested (Supplementary Figures 3A,B).

### Effect of *Trichoderma* Isolates on the Water Deficit Stress Response of ‘Roma Organic’ Tomato

The water deficit stress regimen imposed was effective at reducing water availability to the treated plants (Supplementary Figure 4). Irrigation significantly affected the performance of tomato genotype ‘Roma Organic’ (Supplementary Table 3) with the aboveground and belowground biomass of continuously irrigated plants (43.08 ± 1.18; 12.54 ± 3.83, respectively) higher than biomass of those under the water deficit stress regimen (26.85 ± 1.17; 9.62 ± 3.83, respectively).

**FIGURE 3:**
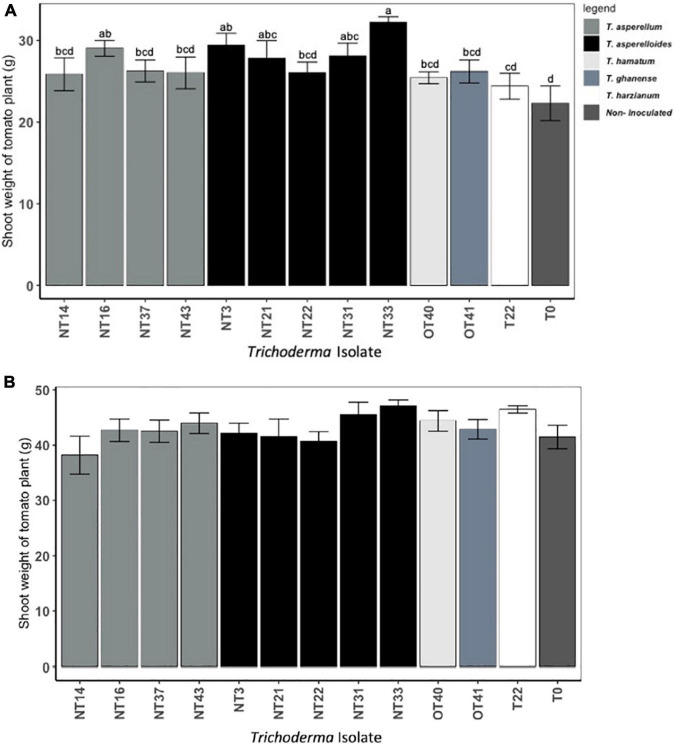
Effect of *Trichoderma* isolates on shoot weight of Roma tomato subjected to: **(A)** water deficit stress, **(B)** continuous irrigated conditions. The water deficit stress was imposed by withholding the irrigation for 5 days. Thereafter, the stressed plants were irrigated again for a 7-day recovery period prior to harvest. The shoot fresh weights per plant were recorded. Values are means ± SE of three trials each with four replicates. Isolates with different superscripts are significantly different according to Fisher LSD test (*P* < 0.05). Bar colors represent the species of tested isolates.

**FIGURE 4:**
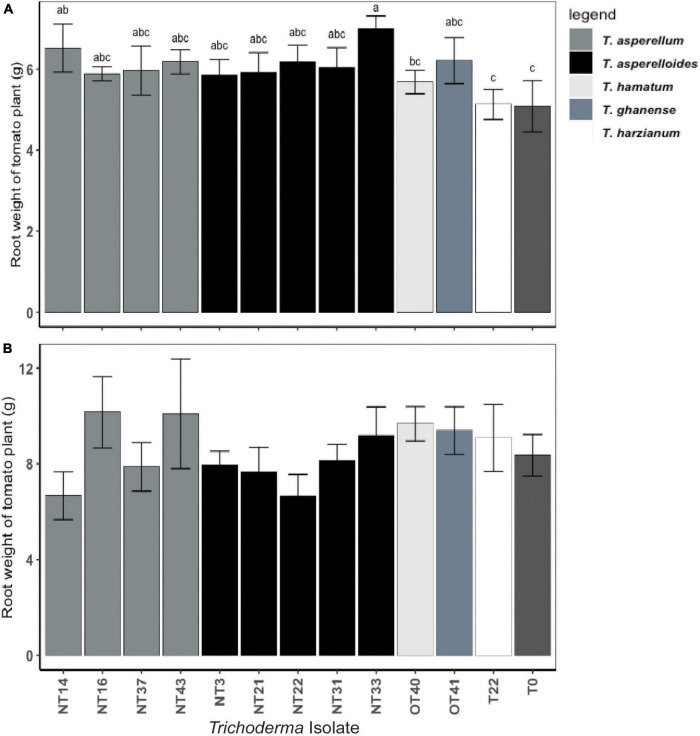
Effect of *Trichoderma* isolates on root weight of Roma tomato subjected to: **(A)** water deficit stress, **(B)** continuous irrigated conditions. Plants were harvested simultaneously with their counterparts subjected to a water deficit stress regimen 32 days after transplanting. The root fresh weights per plant were recorded. Values are means ± SE of three trials each with four replicates. Isolates with different superscripts are significantly different according to Fisher LSD test (*P* < 0.05). Bar colors represent the species of tested isolates.

Shoot and root weights were significantly affected by *Trichoderma* inoculation (Figures 3A,B and Supplementary Table 3). However, as indicated by parsed analysis of the isolate X irrigation interaction (i.e., the “slices” evaluating isolate differences at each level of irrigation), the significance of *Trichoderma* inoculation on shoot biomass was evinced primarily among plants under the water deficit stress regimen (*p* < 0.05; Figure 3A) whereas the effects of inoculation on shoot biomass in continuously irrigated plants were insignificant (*p* = 0.14; Figure 3B). Under water deficit stress conditions, the plants inoculated with NT3, NT16, NT21, NT31, and NT33 accumulated significantly greater aboveground biomass (31–44% higher shoot weight) as compared to the non-inoculated control (T_0_) (Figure 3A). The fresh shoot weight of plants inoculated with commercial *Trichoderma* T22 was not statistically different from that of the non-inoculated control (T_0_) plants. Plants inoculated with NT14, NT16, and NT33 accumulated significantly greater root biomass (20–25% higher root weight) compared to non-inoculated control (T_0_) and commercial *Trichoderma* T22-inoculated plants (Figure 4A). Under irrigation, inoculation with most of the selected *Trichoderma* isolates did not significantly affect the mean shoot weight and root weight of tomato plants compared with those of the non-inoculated control (Figures 3B, 4B and Supplementary Table 3).

### Effect of Selected *Trichoderma* Isolates on the Growth Parameters of Tomato Genotypes Differing in Water Deficit Stress Tolerance

The water deficit regimen curtailed the accumulation of above-ground biomass in water-stressed plants of all three genotypes compared to their continuously irrigated counterparts (Tables 1, 2 and Supplementary Table 4). While evaluating the effects of *Trichoderma* isolates and genotypes at each level of the irrigation treatments (water deficit stress and irrigation conditions), the significance of *Trichoderma* inoculation on growth parameters were evinced in three different genotypes of tomato (*p* < 0.001, Supplementary Table 4).

**TABLE 1:** Effect of four *Trichoderma* isolates on fresh shoot weight, fresh root weight, and number of leaves on three tomato genotypes under water deficit stress conditions^x^.

Genotypes^y^/ Isolates^z^	Shoot weight per plant (g)^x^	Root weight per plant (g)^x^	Number of leaves per plant^x^
NT3 × JF	58.9 ± 1.2 cd	13.4 ± 0.6 ef	9.4 ± 0.4 a–d
NT16 × JF	56.9 ± 1.6 c–e	14.0 ± 0.6 de	7.9 ± 0.5 e–g
NT33 × JF	68.3 ± 1.6 a	16.1 ± 1.1 cd	9.1 ± 0.2 b–e
T22 × JF	52.0 ± 1.6 ef	12.0 ± 0.5 ef	7.8 ± 0.5 fg
T_0_ × JF	49.7 ± 1.2 f	11.3 ± 0.6 f	8.3 ± 0.5 e–g
NT3 × RO	56.3 ± 1.6 de	19.6 ± 1.2 ab	8.5 ± 0.4 c–f
NT16 × RO	54.6 ± 2.2 d–f	19.5 ± 0.8 ab	7.9 ± 0.2 fg
NT33 × RO	64.6 ± 2.6 ab	19.2 ± 0.9 ab	8.6 ± 0.5 c–f
T22 × RO	49.7 ± 1.9 f	19.3 ± 0.8 ab	7.2 ± 0.3 g
T_0_ × RO	49.3 ± 2.6 f	18.2 ± 1.0 bc	7.1 ± 0.4 g
NT3 × PB	61.9 ± 1.9 bc	20.1 ± 1.2 ab	10.0 ± 0.4 ab
NT16 × PB	66.2 ± 2.6 ab	20.4 ± 1.2 ab	10.6 ± 0.3 a
NT33 × PB	65.0 ± 2.3 ab	20.8 ± 1.0 a	9.6 ± 0.6 a–c
T22 × PB	54.3 ± 1.7 d-f	19.4 ± 0.9 ab	8.8 ± 0.3 b–f
T_0_ × PB	59.0 ± 1.7 cd	20.1 ± 0.9 ab	9.7 ± 0.4 a–c
*p*-value (Genotype × Isolates)	<0.0001	<0.0001	<0.0001

*^x^The shoot weight, root weight, and number of leaves per plant were recorded at harvest (32 days after transplanting). Values are means ± SE of two trials each with nine replicates. Means in a column followed by different letters are significantly different according to Fisher LSD test (P < 0.05).*

*^y^Tomato genotypes: JF, ‘Jaune Flamme’; RO, ‘Roma Organic’; and PB, Punta Banda’ subjected to drought and recovery regimens. The drought stress was imposed by withholding irrigation for 5 days. Thereafter, the stressed plants were irrigated again for a 7-day recovery period prior to harvest.*

*^z^Trichoderma isolates NT3 = T. asperelloides, NT16 = T. asperellum, NT33 = T. asperelloides, T22 = T. harzianum, T_0_ = non-inoculated control.*

**TABLE 2:** Effect of four *Trichoderma* isolates on fresh shoot weight, fresh root weight, and number of leaves on three tomato genotypes under irrigated conditions^x^.

Genotypes^y^/ Isolates^z^	Shoot weight per plant (g)	Root weight per plant (g)	Number of leaves per plant
NT3 × JF	84.4 ± 2.1 a–c	14.8 ± 0.9 fg	13.0 ± 0.4 a–c
NT16 × JF	83.0 ± 2.9 a–d	16.1 ± 1.0 f	13.0 ± 0.4 a–c
NT33 × JF	87.9 ± 1.4 a	15.8 ± 0.9 fg	13.1 ± 0.6 a–c
T22 × JF	76.0 ± 2.5 ef	13.6 ± 0.8 g	12.1 ± 0.5 cd
T_0_ × JF	77.2 ± 2.6 ef	13.9 ± 0.6 fg	12.9 ± 0.7 bc
NT3 × RO	78.6 ± 1.9 de	22.2 ± 1.2 de	9.9 ± 0.3 f
NT16 × RO	76.4 ± 1.8 ef	27.6 ± 1.7 a	11.3 ± 0.4 de
NT33 × RO	78.8 ± 1.9 de	25.4 ± 1.5 ab	10.3 ± 0.3 ef
T22 × RO	75.4 ± 1.9 ef	24.1 ± 1.1 b–d	10.4 ± 0.4 ef
T_0_ × RO	69.9 ± 3.9 g	23.9 ± 1.4 b–e	12.2 ± 0.4 cd
NT3 × PB	79.4 ± 2.8 b–e	22.9 ± 1.4 c–e	13.8 ± 0.6 ab
NT16 × PB	79.2 ± 2.7 c–e	23.3 ± 1.3 b–e	13.8 ± 0.7 ab
NT33 × PB	84.6 ± 2.9 ab	24.6 ± 1.1 bc	14.2 ± 0.8 a
T22 × PB	72.0 ± 2.9 fg	21.8 ± 1.3 e	12.2 ± 0.7 cd
T_0_ × PB	75.6 ± 2.7 ef	22.6 ± 0.9 c–e	13.8 ± 0.6 ab
*p* value (Genotype × Isolates)	<0.0001	<0.0001	<0.0001

*^x^The shoot weight, root weight, and number of leaves per plant were recorded at harvest (32 days after transplanting). Values are means ± SE of two experimental runs each with nine replicates. Means in a column followed by different letters are significantly different according to Fisher LSD test (P < 0.05).*

*^y^ Tomato genotypes: JF, ‘Jaune Flamme’; RO, ‘Roma Organic’; and PB, ‘Punta Banda’.*

*^z^Trichoderma isolates NT3 = T. asperelloides, NT16 = T. asperellum, NT33 = T. asperelloides, T22 = T. harzianum, T_0_ = non-inoculated control.*

**FIGURE 5:**
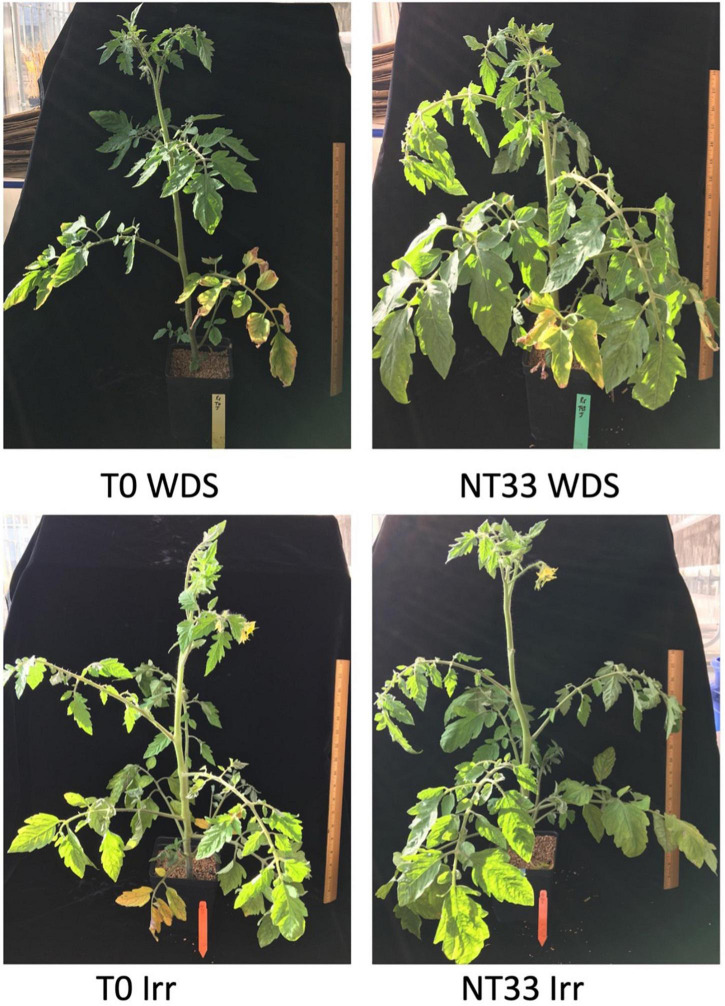
Example ‘Jaune Flamme’ tomato plants grown under different Trichoderma inoculation and water deficit stress treatments. NT33, *T. asperelloides* isolate. T0WDS, non-inoculated water stressed plants; NT33WDS, NT33 inoculated water stressed plants; T0 Irr, non-inoculated irrigated plants; NT33 Irr, NT33 inoculated irrigated plants. Images were obtained after recovery from water deficit stress.

Under water deficit stress conditions, ‘Jaune Flamme’ plants inoculated with NT3 and NT33 had significantly higher shoot weight (about 18 and 37%, respectively) than non-inoculated control (T_0_ × JF) and commercial *Trichoderma* inoculated plants (T22 × JF) (Table 1 and Figure 5). ‘Jaune Flamme’ plants inoculated with NT16 had significantly higher shoot weight (14%) compared to non-inoculated control plants (T_0_ × JF). In ‘Roma Organic,’ plants application of *Trichoderma* isolates NT3 and NT33 significantly increased the shoot biomass (14 and 30% respectively) as compared to non-inoculated control (T_0_ × RO) and T22 inoculated tomato plants (T22 × RO) (Table 1). Similarly, inoculation of ‘Punta Banda’ plants with NT16 and NT33 resulted in significantly greater shoot weight (12%) compared to non-inoculated control plants (T_0_ × PB) (Table 1). Only ‘Jaune Flamme’ plants inoculated with NT33 had significantly higher root weight (34–48%) compared to commercial *Trichoderma* T22-inoculated (T22 × JF) and non-inoculated control plants (T_0_ × JF) under water deficit stress conditions (Table 1). ‘Jaune Flamme’ plants inoculated with NT3 maintained significantly higher leaf numbers (13%) than non-inoculated controls (T_0_ × JF) (Table 1). In ‘Roma Organic,’ plants inoculated with NT3 and NT33 maintained significantly higher leaf numbers (20%) than non-inoculated controls (T_0_ × RO) or commercial *Trichoderma* inoculated plants (T22 × RO) (Table 1). However, *Trichoderma* inoculation had no effect on root weight and number of leaves per plant in ‘Punta Banda’ compared to the non-inoculated control (T_0_ × PB) plants under water deficit stress conditions (Table 1).

Under irrigated conditions, inoculation with the *Trichoderma* isolates NT16, NT3, and NT33 resulted in higher shoot biomass, 9–13% in ‘Jaune Flamme’ and 9 to 12% in ‘Roma Organic’ plants compared to their non-inoculated control plants (T_0_ × JF and T_0_ × RO, respectively) (Table 2). Similarly, in ‘Punta Banda,’ *Trichoderma* isolate NT33 significantly increased the shoot weight by 12% as compared to the non-inoculated control plants (T_0_ × PB). ‘Roma Organic’ plants inoculated with NT16 had significantly higher root biomass than non-inoculated control plants (T_0_ × PB) but the root weights of *Trichoderma* inoculated plants were not significantly different from the non-inoculated control in other tomato genotypes (Table 2). *Trichoderma* inoculation of ‘Jaune Flamme’ plants had no effect on the number of leaves per plant, but T22-inoculated ‘Punta Banda’ and ‘Roma Organic’ plants inoculated with T22, NT3, or NT33 produced approximately 1-2 fewer leaves in comparison to non-inoculated controls (Table 2 and Supplementary Table 5).

### Plant Physiological Responses to Water Deficit Stress and *Trichoderma* Inoculation

Water deficit stress significantly reduced the relative water content percentage (RWC) in the tomato leaves (*p* < 0.001; Tables 3, 4 and Supplementary Table 5). Application of NT33 resulted in significantly higher RWC (16%) in the genotype ‘Jaune Flamme’ compared to non-inoculated controls (T_0_ × JF) and *T. harzianum* T22 inoculated tomato plants (T22 × JF), whereas in ‘Roma Organic’ plants there was 36% more RWC in NT3 inoculated plants compared to non-inoculated controls (T_0_ × RO) and T22 inoculated tomato (T22 × RO) plants (Table 3). Similarly, the NT33 application significantly increased the chlorophyll content in ‘Jaune Flamme’ and ‘Roma Organic’ plants by (11 and 12%, respectively) compared to non-inoculated control plants (Table 3). In addition, ‘Jaune Flamme’ plants inoculated with NT3 and T22, and ‘Roma Organic’ plants inoculated with NT3 and NT16 had significantly higher chlorophyll content (4.4 and 4.5%, respectively) compared to non-inoculated control plants (T_0_ × JF and T_0_ × RO) (Table 3). However, *Trichoderma* treatment had no significant effect on RWC content and chlorophyll content in ‘Punta Banda’ genotype compared to non-inoculated control under water deficit stress conditions.

**TABLE 3:** Effect of four *Trichoderma* isolates on chlorophyll content, relative water content, stomatal conductance and net photosynthesis on three tomato genotypes under water deficit stress conditions^x^.

Genotypes^y^/ Isolates^z^	Relative water content (%)	Chlorophyll content (SPAD)	Stomatal conductance [mM(H2O)m^–2^s^–1^]	Photosynthesis [μM(CO_2_)m^–2^s^–1^]
NT3 × JF	60.9 ± 2.2 ab	42.6 ± 0.6 d	25.4 ± 2.7 d–f	1.96 ± 1.3 a
NT16 × JF	59.2 ± 0.4 a–c	41.7 ± 0.8 d-f	23.7 ± 1.2 ef	1.52 ± 0.8 ab
NT33 × JF	64.7 ± 4.0 a	45.1 ± 0.7 ab	27.1 ± 2.1 b-e	1.62 ± 0.4 ab
T22 × JF	55.4 ± 5.0 b–d	42.6 ± 0.5 d	26.3 ± 0.5 c–f	–0.83 ± 0.2 c–f
T_0_ × JF	55.7 ± 1.7 b–d	40.8 ± 0.4 ef	23.2 ± 1.0 f	–1.33 ± 0.9 ef
NT3 × RO	63.9 ± 2.8 a	42.9 ± 0.9 cd	25.8 ± 2.1 c–f	–1.01 ± 0.6 d–f
NT16 × RO	46.8 ± 2.1 e	42.2 ± 0.7 de	27.1 ± 0.3 b–e	–1.68 ± 1.2 f
NT33 × RO	51.4 ± 1.3 de	45.5 ± 0.9 ab	34.1 ± 3.6 a	–0.23 ± 0.6 c–f
T22 × RO	49.6 ± 2.5 de	41.6 ± 0.8 d–f	28.3 ± 3.6 b–d	0.52 ± 2.0 a–d
T_0_ × RO	46.9 ± 1.5 e	40.4 ± 0.8 f	26.1 ± 1.3 c–f	–0.74 ± 0.8 c–f
NT3 × PB	53.2 ± 2.7 c–e	44.6 ± 0.7 a–c	28.3 ± 1.4 b–d	1.79 ± 0.7 ab
NT16 × PB	53.6 ± 1.9 c–e	46.1 ± 0.8 a	30.1 ± 3.2 b	0.52 ± 1.3 a–d
NT33 × PB	55.6 ± 2.7 b–d	45.7 ± 0.7 ab	25.7 ± 2.1 c–f	1.64 ± 0.9 ab
T22 × PB	54.6 ± 2.5 b–d	43.3 ± 0.7 cd	29.0 ± 2.8 bc	1.05 ± 0.6 a–c
T_0_ × PB	53.9 ± 3.2 b–e	44.4 ± 0.8 a–c	24.3 ± 5.5 ef	0.11 ± 2.2 b–e
*p*-value (Genotype × Isolates)	0.05	<0.0001	0.03	0.07

*^x^Chlorophyll content was recorded two days after recovery, relative water content (%), stomatal conductance, and photosynthesis were recorded at 5th day of water deficit stress. Values are means ± SE of two experimental runs each with five replicates for chlorophyll content. For other physiological parameters, values are means ± SE of one experimental trial each with three replicates. Means in a column followed by different letters are significantly different according to Fisher LSD test (P < 0.05).*

*^xy^Three tomato genotypes: JF, ‘Jaune Flamme’; RO, ‘Roma Organic’; and PB, ‘Punta Banda’.*

*^xz^Four Trichoderma isolates; NT3 = T. asperelloides, NT16 = T. asperellum, NT33 = T. asperelloides, T22 = T. harzianum -T22, T_0_ = non-inoculated control.*

**TABLE 4:** Effect of four *Trichoderma* isolates on chlorophyll content, relative water content, stomatal conductance, and net photosynthesis on three tomato genotypes under irrigated conditions^x^.

Genotypes^y^/ Isolates^z^	Relative water content (%)	Chlorophyll content (SPAD)	Stomatal conductance [mM(H2O)m^–2^s-]	Photosynthesis [μM(CO_2_)m^–2^s^–^]
NT3 ×JF	94.5 ± 0.7	41.9 ± 0.5 d–f	771.4 ± 1.4	7.5 ± 1.3
NT16 × JF	94.8 ± 1.2	42.9 ± 0.7 c–e	644.0 ± 2.5	7.3 ± 1.7
NT33 × JF	93.0 ± 0.7	43.9 ± 0.3 bc	582.8 ± 0.9	7.2 ± 1.5
T22 × JF	93.8 ± 0.8	42.2 ± 0.5 c-f	456.3 ± 1.5	7.3 ± 1.9
T_0_ × JF	92.8 ± 1.4	42.7 ± 0.7 c–e	698.0 ± 1.7	6.9 ± 1.9
NT3 × RO	94.7 ± 0.7	42.1 ± 0.7 d–f	774.0 ± 2.5	7.1 ± 1.6
NT16 × RO	93.4 ± 1.3	43.4 ± 0.5 b–e	739.3 ± 1.5	7.5 ± 1.2
NT33 × RO	92.8 ± 0.7	43.5 ± 0.5 b–d	614.3 ± 1.2	6.9 ± 1.3
T22 × RO	93.5 ± 0.7	41.7 ± 0.6 ef	440.3 ± 1.8	7.7 ± 1.7
T_0_ × RO	94.1 ± 1.5	40.9 ± 0.5 f	702.0 ± 1.4	7.5 ± 1.7
NT3 × PB	93.9 ± 1.1	47.7 ± 0.4 a	666.0 ± 1.6	7.5 ± 3.0
NT16 × PB	94.3 ± 1.1	47.0 ± 0.5 a	790.7 ± 1.2	7.8 ± 1.5
NT33 × PB	92.5 ± 0.8	47.6 ± 0.6 a	569.7 ± 1.7	7.3 ± 1.4
T22 × PB	93.7 ± 0.8	45.0 ± 0.6 b	380.3 ± 2.0	7.6 ± 2.1
T_0_ × PB	92.8 ± 1.4	46.9 ± 0.5 a	606.7 ± 0.8	8.3 ± 2.9
*p*-value (Genotype × Isolates)	1.0	<0.0001	0.8	1.0

*^x^Chlorophyll content, relative water content (%), stomatal conductance and photosynthesis of non-stressed or irrigated plants were recorded simultaneously with their counterparts subjected to water deficit stress. Values are means ± SE of two experimental runs each with five replicates for chlorophyll content. For other physiological parameters, values are means ± SE of one experimental trial each with three replicates. Means in a column followed by different letters are significantly different according to Fisher LSD test (P < 0.05).*

*^xy^Three tomato genotypes: JF, ‘Jaune Flamme’; RO, ‘Roma Organic’; and PB = ‘Punta Banda’.*

*^xz^Four Trichoderma isolates; NT3 = T. asperelloides, NT16 = T. asperellum, NT33 = T. asperelloides, T22 = T. harzianum -T22, T_0_ = non-inoculated control.*

Physiological parameters such as photosynthetic rate and stomatal conductance were significantly reduced under water deficit stress condition compared to irrigated conditions (*p* < 0.001; Tables 3, 4 and Supplementary Table 6). Plants inoculated with *Trichoderma* NT33 had significantly higher stomatal conductance in ‘Jaune Flamme’ and ‘Roma Organic’ plants by 18.8 and 39%, respectively compared to non-inoculated control plants under water deficit stress conditions. ‘Punta Banda’ plants inoculated with NT3 and NT16 had significantly higher (16–23%) stomatal conductance compared to non-inoculated control (T_0_ × PB). Similarly, Nepalese *Trichoderma* inoculation resulted in higher photosynthetic rate in ‘Jaune Flamme’ plants than non-inoculated control and T22 inoculated plants (Table 3). However, the effect of *Trichoderma* application on the photosynthetic health of the other two genotypes were not significantly different from non-inoculated controls under water deficit stress conditions. Under irrigated conditions, the inoculation of ‘Jaune Flamme,’ ‘Roma Organic,’ and ‘Punta Banda’ plants with *Trichoderma* isolates NT3, NT16, and NT33 did not significantly change the measured physiological parameters when compared to T22 inoculated or the non-inoculated control plants (Table 4 and Supplementary Table 6).

## Discussion

The application of biological agents like water deficit-tolerant *Trichoderma* could be a low-cost, environmentally friendly, and sustainable near-term solution to mitigate drought stress (Shukla et al., 2012; Brotman et al., 2013). However, specific fungal isolate-plant cultivar relationships are complex, and the mechanisms of drought tolerance induced in the plant by *Trichoderma* colonization are not well-understood. Herein, we report the evaluation of novel *Trichoderma* isolates collected from different agroecological regions of Nepal for their ability to proliferate under osmotic stress and then to mitigate stress responses in tomato genotypes during and after exposure to water deficit conditions. We had a unique opportunity to test isolates from highly diverse ecological niches ranging in mean annual precipitation from <200 to 5,000 mm, in elevation above sea level from 58 to 8,848 m, and temperature extremes from –12°C to 43.3°C (Karki et al., 2016; Khadka et al., 2022).

Supplementation of growth medium with PEG is designed to measure the effect of water potential on the growth of fungi (Jin et al., 1991; Aujla and Paulitz, 2017). We screened 44 isolates and identified some isolates which exhibited the ability to survive and sporulate under different osmotic stress conditions (Figures 2A–C and Supplementary Table 2). The potential efficacy of these isolates to mitigate water deficit stress *in planta* was ascertained in greenhouse experiments, in which tomatoes inoculated with NT16, NT3, NT33 and subjected to water deficit stress amassed significantly greater growth parameters than the non-inoculated water deficit stressed control plants in different tomato genotypes (Figure 3A and Table 1). Similarly, other researchers have successfully employed various *in vitro* culture techniques to identify isolates of *Trichoderma* (Shukla et al., 2012; Poosapati et al., 2014; Khoshmanzar et al., 2020) or other soil microbiota (Mhadhbi et al., 2011; Subramanian et al., 2016) that conferred tolerance to abiotic stressors, such as osmotic (salt, drought) or temperature (heat, cold) stress.

Microorganisms collected from environmentally diverse sites (arid, saline, alpine regions) are reservoirs of potential traits that may benefit plant hosts when grown under the abiotic stress conditions from where they were isolated (Subramanian et al., 2016; Giauque et al., 2019). Among the six *Trichoderma* isolates from Nepal that consistently performed in the top third of the collection in the *in vitro* assay, NT3 was isolated in Jumla, the driest site sampled (766 mm/year precipitation; Supplementary Tables 1, 2 and Supplementary Figure 1). Isolates NT31, NT33, and NT37 were obtained from Banke, another dry site (1175 mm annual precipitation with 85% of the rainfall accumulated during a three-month monsoon season). Conversely, isolates NT10, NT19, NT23, and NT27 ranking in the lower third of the collection for growth under water deficit conditions were collected at sites receiving 1993–2329 mm annual precipitation, from 160 to 200% greater than that received at Jumla. To our knowledge, site-specific *Trichoderma* adaptation to stress environments, either as free-living organisms or in mutualistic relationships, has yet to be reported. Adaptation to mutualism as a means of survival under harsh environmental conditions may be predicated upon co-evolution involving heritable genetic variation in both plants and microorganisms that promote colonization as well as abiotic stress responses (Hoeksema, 2010). Kubicek et al. (2019) characterized *Trichoderma* as an “environmental opportunist and generalist” that can rapidly evolve to thrive in new or stressful environments as a free-living organism or a plant symbiont. The fungi can produce the asexual conidiospores and chlamydospores that can survive under severe environmental conditions. Therefore, it is plausible that specific *Trichoderma* isolates in this study, most notably NT33, are effective at ameliorating water deficit stress in tomatoes because they evolved in drier regions of Nepal. The evolution of these and other effective isolates may also have been influenced by micro-climatic conditions not examined during the collection process.

Water deficit stress significantly inhibits crop productivity and quality. *Trichoderma* inoculation could ameliorate the negative effect of water deficit stress and facilitate growth promotion in plants (De Palma et al., 2016; Bashyal et al., 2021). Mastouri et al. (2010, 2012) demonstrated the effects of *Trichoderma* inoculation on plant performance to be apparent only in stressful environments. In their studies, inoculation of tomato seed with T22 improved germination and other growth parameters when grown at water deficit conditions. Other researchers have uncovered similar trends among treatments and controls, suggesting significant *Trichoderma* effects under different biotic or abiotic stress conditions (Donoso et al., 2008; Lombardi et al., 2018). Our results support these findings in the first greenhouse experiment where shoot weights of ‘Roma Organic’ tomato genotype inoculated with five *Trichoderma* isolates belonging to two different species *T. asperellum* and *T. asperelloides* were higher than those of controls when subjected to the water deficit regimen (Figure 3A). However, in the *Trichoderma* × Tomato Genotype experiment, there was a significant effect of *Trichoderma* inoculation on growth parameters of tomato plants under both irrigated and water deficit stress conditions (Tables 1, 2), supporting *Trichoderma*’s role as growth promoting agent.

Genotypes of the plant and symbiont are essential factors determining the level of the beneficial effect of *Trichoderma* inoculation, even for established and commercial isolates of *Trichoderma* like T22 and the *T. virens* strain G-41 (Harman et al., 2004; Prashar and Vandenberg, 2017). *Trichoderma*-cultivar specificity has been studied most extensively upon exposure to biotic stresses, but our study documents this relationship under the abiotic stress condition (water deficit stress) as well. We conducted the greenhouse trial with three genotypes of tomato differing in their drought tolerance capacity. Since plant- microbe interaction is host specific, it is important to identify the isolates that are effective across wide range of crops. In our study, NT33 generally outperformed the other Nepalese *Trichoderma* isolates and the commercial *Trichoderma* T22 when inoculated in the different tomato genotypes under both water deficit stress and non-stress conditions. We also found that the overall performance of the selected *Trichoderma* isolates was better in drought susceptible genotypes ‘Jaune Flamme’ and ‘Roma Organic’ as compared to drought tolerant ‘Punta Banda’ (Tables 1–4). Prashar and Vandenberg (2017) exposed 23 wild and cultivated lentil genotypes colonized with either commercial *Trichoderma* strain T22 or G41 to the root rot pathogen *Aphanomyces euteiches*. Responses to the pathogen and overall plant performance were strongly governed by the origin and genetic makeup of the lentil genotype. Similarly, using *Trichoderma* commercial strains P1 (*T. atroviride*) and T22 and five tomato genotypes, Tucci et al. (2011) demonstrated significant variability in performance (negative, neutral, positive) among inoculated pairs vs. controls. These authors introduced the feasibility of breeding plant genotypes to be specifically responsive to common commercial *Trichoderma* isolates.

Water deficit stress stimulates different physiological responses such as stomatal closure, repression of photosynthesis, activation of respiration, and reduction of cell growth in the plants. The plants inoculated with *Trichoderma* isolates can enhance water-deficit tolerance by improving root development, regulation of drought-induced changes in the stomatal opening, photosynthesis, and chlorophyll content in leaf (Contreras-Cornejo et al., 2009; Brotman et al., 2013; Guler et al., 2016). Our study also found that the net photosynthetic rate and stomatal conductance in the tomato leaves were less affected by water deficit stress in the NT33 inoculated tomato plants compared to non-inoculated control (Table 3). In addition, NT33 and NT3 increased the chlorophyll content in the drought susceptible genotypes as compared to non-inoculated controls, which is an important pigment essential for absorbing light during photosynthetic process (Table 3). Bashyal et al. (2021) reported that genes related to the photosynthesis process, such as those coding for photosystem I subunits, photosystem II core complex proteins, osmotin like proteins, and stomatal development were upregulated in the *Trichoderma* inoculated plants as compared to non-inoculated controls. Water deficit exposed plants inoculated with *Trichoderma* exhibit improved physiological health (Shukla et al., 2012; Harman et al., 2019; Khoshmanzar et al., 2020), similar to our observation in NT33-colonized tomato plants. It has been reported that the plants inoculated with *Trichoderma* species increase the accumulation of different metabolites like phenolics, flavonoids, and proline which increase antioxidant activity, protect photosynthetic pigments, and photosynthetic pathways from photooxidative damage (Mona et al., 2017; Scudeletti et al., 2021; Tripathi et al., 2021). The earliest response of plants to drought is considered to be due to an increase in abscisic acid content and regulates stomatal movements and helps to maintain water balance. Martinez-Medina et al. (2013) reported that *Trichoderma harzianum* colonized plants displayed higher expression levels of the ABA-responsive marker gene involve in different defense-related pathways compared to non-inoculated control plants.

Relative water content is an important indicator of the physiological water status of the leaf. It indicates the appropriate water status of plant in terms of cellular hydration and is used as a selection criterion for drought tolerance in different crops (Teulat et al., 1997). Under water deficit stress conditions, the RWC% in the plants is significantly reduced; however, the *Trichoderma* colonization augmented the relative water status in the leaves (Bae et al., 2009; Guler et al., 2016). In our study NT33 and NT3 inoculation increased the relative water content in the leaf of Jaune Flamme’ and ‘Roma Organic’ plants and allowed them to defend against the abiotic stress (Table 3). *Trichoderma* spp. can improve the drought avoidance mechanism in plants through morphological adaptation by increasing root growth and also changing root architecture in many crops, thereby increasing the plant yield under the water deficit stress condition (Contreras-Cornejo et al., 2009; Meng et al., 2019). Some *Trichoderma* species secrete auxin (indole acetic acid) and other indole derivatives like nitrilase, that induce the lateral root growth and increase the root surface area (Hermosa et al., 2012). In this study we found that the ‘Jaune Flamme’ plants inoculated with NT33 had higher root biomass compared to T_0_ (Table 1) under water deficit stress condition and also increases the lateral root number (Rawal, 2021) that could have increased the water status in the plant.

The salient results of this study are as follows: (1) We demonstrated the ability of *Trichoderma* isolates to reduce water deficit stress in tomato, which may provide a grower-friendly strategy for crop improvement and crop protection. (2) We identified superior strains from among a novel collection of 41 *Trichoderma* isolates collected from diverse ecological niches in Nepal with the ability to thrive under osmotic stress and to improve the horticultural performance of inoculated tomatoes when cultured in the greenhouse under water deficit stress conditions. (3) We ascertained that the best performing isolates were collected in dry to relatively dry regions of the country, suggesting adaptations to mutualism predisposing them to confer water deficit tolerance to the plants they colonized. This potential adaptation response to environmental conditions may have significant implications for the acquisition of *Trichoderma* strains capable of enhancing crop performance under specific biotic and abiotic stress conditions. (4) We validated plant cultivar-*Trichoderma* isolate specificity in greenhouse experiments with inoculated tomato plants differing in water stress tolerance. The improvement in horticultural performance was most evident in inoculated, water stress-susceptible tomato genotypes. (5) We distinguished NT33 (*T. asperelloides*) isolate for its ability to deliver consistent performance advantages to its tomato partners suggesting its potential as an element in multi-faceted environmentally sound approaches to manage crop water deficit stress (Figure 5). It is noteworthy that NT33 also consistently outperformed the commercially available T22 (*T. harzianum*) strain suggesting that limits to the potential benefits of *Trichoderma* inoculation under abiotic stress conditions have yet to be reached. Different biochemical and genetic determinants underlying the induction of resistance in the plants are unknown and are currently under investigation. In the future, research should also focus on the mechanisms adopted by these isolates like solubilization and mobilization of nutrients, production of plant growth-promoting hormones, and other secondary metabolites for plant growth promotion and adoption to abiotic stress.

## Data Availability Statement

The original contributions presented in the study are included in the article/Supplementary Material, further inquiries can be directed to the corresponding author/s.

## Author Contributions

RR, JS, and M-SB conceived the project. RR performed most of the experiments, analyzed the data, and wrote the manuscript. SF and DF conceived the experiments on water deficit susceptible tomato genotypes. SF performed those experiments and wrote the corresponding sections. SM provided *Trichoderma* isolates. JS, M-SB, SM, and DF edited the manuscript. All the authors contributed to the article and approved the submitted version.

## Conflict of Interest

The authors declare that the research was conducted in the absence of any commercial or financial relationships that could be construed as a potential conflict of interest.

## Publisher’s Note

All claims expressed in this article are solely those of the authors and do not necessarily represent those of their affiliated organizations, or those of the publisher, the editors and the reviewers. Any product that may be evaluated in this article, or claim that may be made by its manufacturer, is not guaranteed or endorsed by the publisher.
