# Supplementary figures and images for: Novel Trichoderma Isolates Alleviate Water Deficit Stress in Susceptible Tomato Genotypes
Source: Front Plant Sci. 2022 May 2;13:869090. doi: 10.3389/fpls.2022.869090 (PMC9108677; doi:10.3389/fpls.2022.869090)

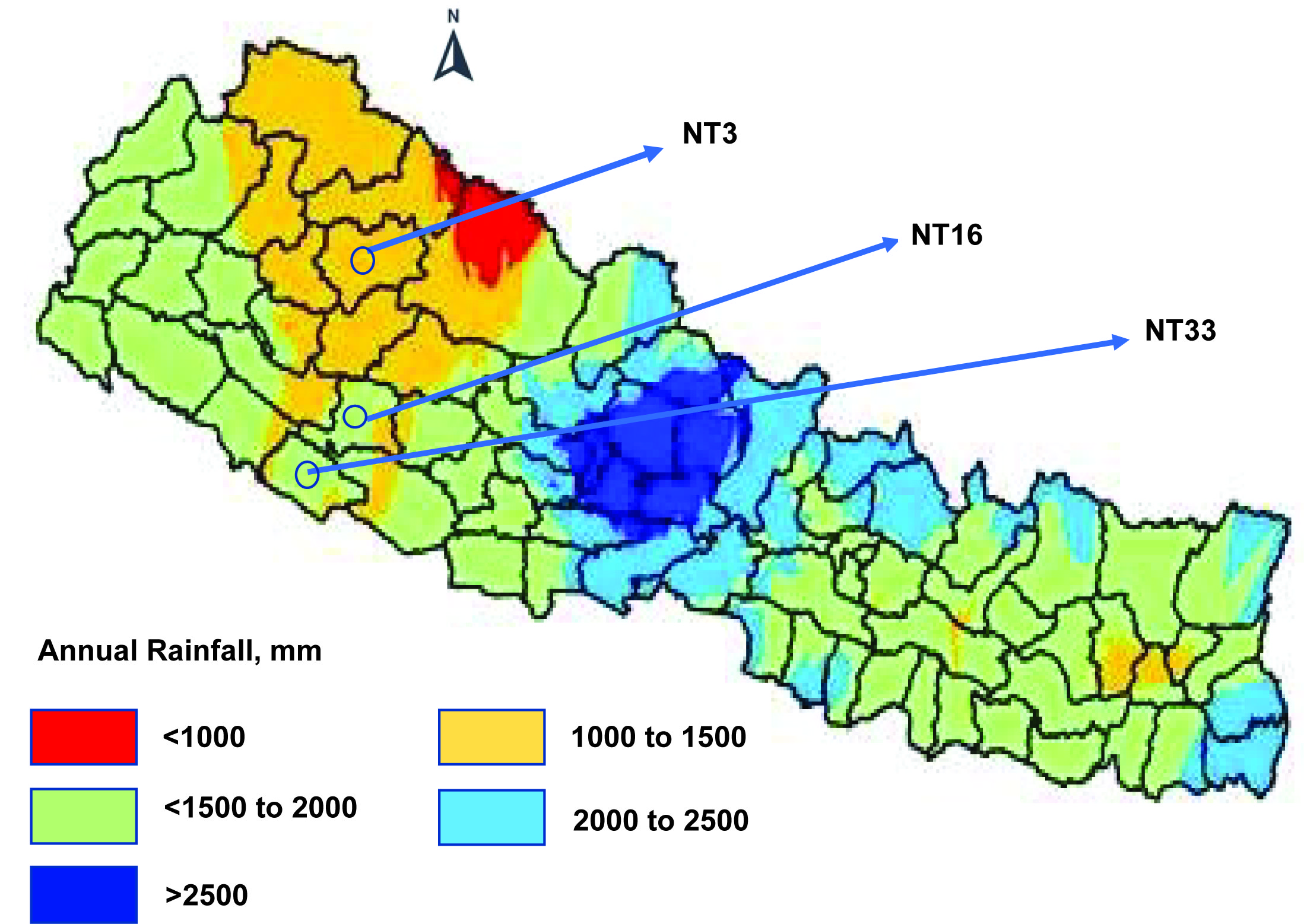

Supplement: Supplementary file 1 [file Image_1.JPEG]

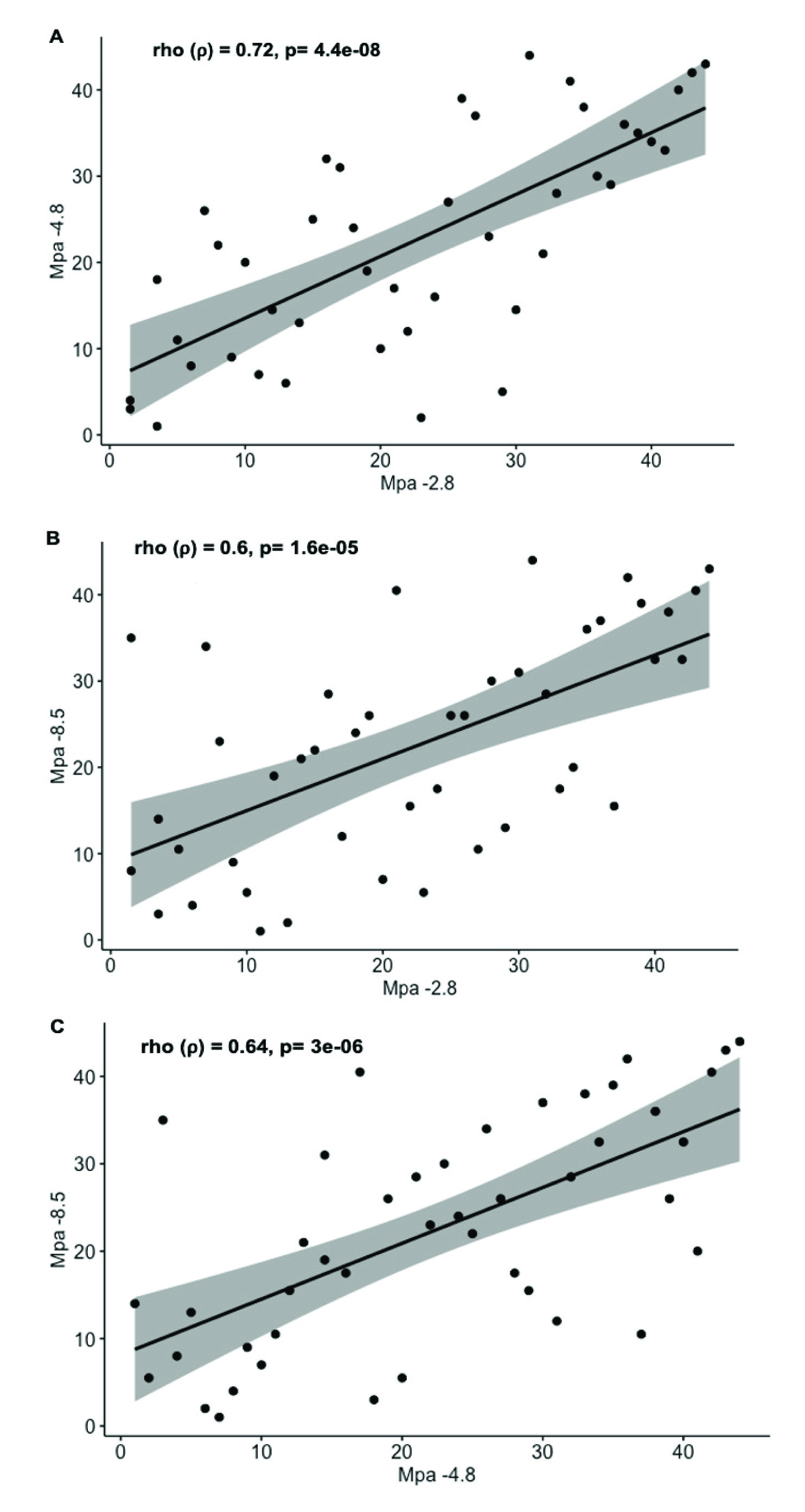

Supplement: Supplementary file 2 [file Image_2.JPEG]

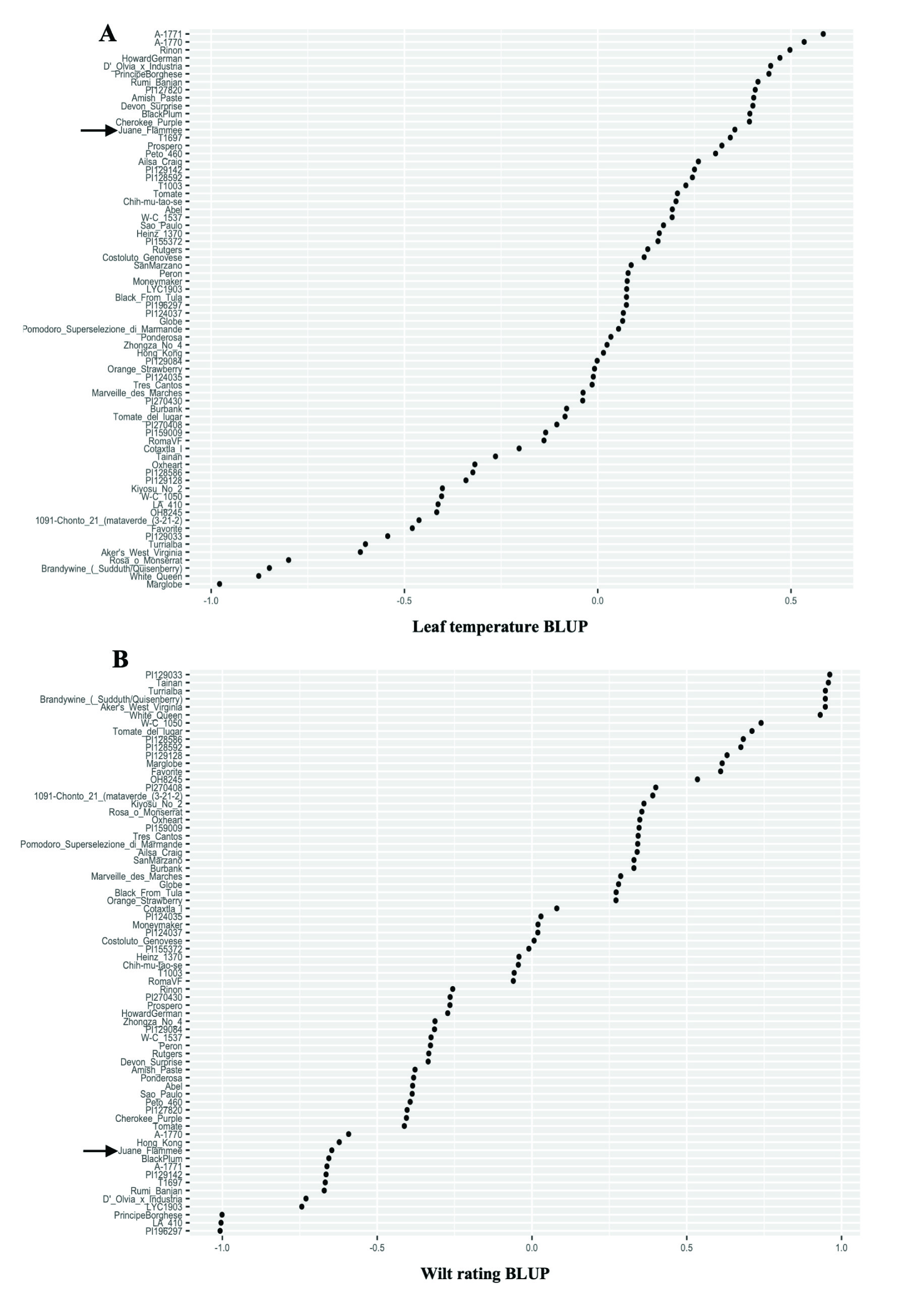

Supplement: Supplementary file 3 [file Image_3.JPEG]

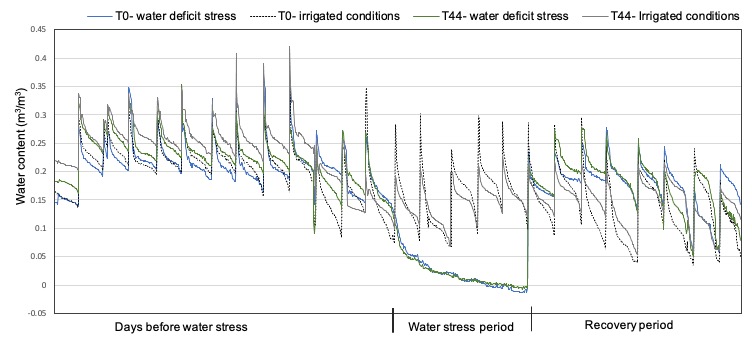

Supplement: Supplementary file 4 [file Image_4.JPEG]
