# Supplementary material for: Novel Trichoderma Isolates Alleviate Water Deficit Stress in Susceptible Tomato Genotypes
Source: Front Plant Sci. 2022 May 2;13:869090. doi: 10.3389/fpls.2022.869090 (PMC9108677; doi:10.3389/fpls.2022.869090)
Supplement: Supplementary file 5 [file Data_Sheet_1.docx]

Supplementary Material

**SUPPLEMENTARY FIGURES**

**Supplementary Fig. S1**. Location of origin of *Trichoderma* isolates NT3, NT16, and NT33 in Nepal and distribution of annual rainfall.

**Supplementary Fig. S2.**Correlation between rank performance of *Trichoderma* isolates cultured in different matric water potential A: Correlation between -2.8 vs. -4.8 Ψ, B: Correlation between -2.8 vs. -8.5, Correlation between -4.8 vs. -8.5 Ψ.

**Supplementary Fig. S3**. Main effects of maximum leaf temperature (higher=more susceptible) and maximum wilt rating (lower=more susceptible) expressed as best linear unbiased predictors (BLUPs) on heirloom and landrace genotypes. (A) Maximum leaf temperature of 70 heirloom and land race genotypes screened for water stress susceptibility. (B) Maximum wilt rating of the 70 genotypes. Arrow indicates ‘Jaune Flamme’.

**Supplementary Fig. S4**. Water content (m^3/^m^3^) in the pots before water stress, during stress and after recovery.

**SUPPLEMENTARY TABLES**

**Supplementary Table S1.**Collection of *Trichoderma* isolates from different agroecological regions of Nepal.

| Isolates^x^ | Area^x^ | TEF/ ITS^x^ | Mycelium color^x^ | Location^xy^ | Elevation  (m)^z^ | Minimum temperature °C^z^ | Maximum temperature °C^z^ | Annual rainfall (mm)^z^ |
| --- | --- | --- | --- | --- | --- | --- | --- | --- |
| NT1 | Chitwan | *T. asperelloides* | Blue | Inner terai | 212 | 15.7 | 29.2 | 1993 |
| NT2 | Palpa | *T. asperellum* | Green | Mid-hill | 1054 | 11.0 | 24.7 | 1724 |
| NT3 | Jumla | *T. asperelloides* | Orange | High- hill | 2362 | 6.3 | 43.3 | 766 |
| NT4 | Palpa | *T. asperellum* | Green | Mid-hill | 1054 | 11.0 | 24.7 | 1724 |
| NT5 | Banke | *T. harziaanum* | Dark red | Terai | 151 | 15.4 | 31.6 | 1175 |
| NT6 | Salyan | *T. asperellum* | Green | Mid-hill | 1579 | 9.0 | 22.0 | 1345 |
| NT7 | Baglung | *T. asperellum* | Green | Mid-hill | 937 | 12.8 | 25.7 | 1793 |
| NT8 | Chitwan | *T. asperelloides* | Green | Inner terai | 212 | 15.7 | 29.2 | 1993 |
| NT9 | Lalitpur | *T. asperelloides* | Green | Mid-hill | 1324 | 9.8 | 23.3 | 1430 |
| NT10 | Chitwan | *T. asperellum* | Green | Inner terai | 212 | 15.7 | 29.2 | 1993 |
| NT11 | Dhankuta | *T. asperellum* | Green | Mid-hill | 1147 | 13.4 | 25.2 | 1002 |
| NT12 | Nawalparasi | *T. asperellum* | Green | Terai | 184 | 16.0 | 29.4 | 2248 |
| NT13 | Achham | *T. harzianum* | Red | Mid-hill | 1361 | 10.5 | 24.1 | 1456 |
| TN14 | Jhapa | *T. asperellum* | Green | Terai | 58 | 10.0 | 30.0 | 2000 |
| NT15 | Mugu | *T. harzianum* | Red | High- hill | 2061 | 8.2 | 21.4 | 873 |
| NT16 | Salyan | *T. asperellum* | Green | Mid-hill | 1579 | 9.0 | 22.0 | 1345 |
| NT17 | Ilam | *T. asperellum* | Green | Mid-hill | 1270 | 12.4 | 22.5 | 1966 |
| NT18 | Salyan | *T. asperellum* | Green | Mid-hill | 1579 | 9.0 | 22.0 | 1345 |
| NT19 | Mangalpur | *T. harzianum* | Red | Inner terai | 212 | 15.7 | 29.2 | 1993 |
| NT20 | Tarahara | *T. asperellum* | Green | Terai | 78 | 16.3 | 29.3 | 1441 |
| NT21 | Rampur | *T. asperelloides* | Orange | Inner terai | 212 | 15.7 | 29.2 | 1993 |
| NT22 | Kaski | *T. asperellum* | Orange | Mid-hill | 854 | 13.1 | 25.3 | 3474 |
| NT23 | Rupandehi | *T. asperellum* | Green | Terai | 177 | 15.2 | 29.6 | 2203 |
| NT24 | Ilam | *T. asperellum* | Green | Mid-hill | 1502 | 10.6 | 20.9 | 2628 |
| NT25 | Dhading | *T. asperellum* | Green | Mid-hill | 606 | 15.1 | 27.6 | 2329 |
| NT26 | Lalitpur | *T. asperellum* | Green | Mid-hill | 1324 | 9.8 | 23.3 | 1430 |
| NT27 | Dhading | *T. asperelloides* | Green | Mid-hill | 606 | 15.1 | 27.6 | 2329 |
| NT28 | Banke | *T. asperellum* | Green | Terai | 151 | 15.4 | 31.6 | 1175 |
| NT29 | Chitwan | *T. asperelloides* | Green | Inner terai | 212 | 15.7 | 29.2 | 1993 |
| NT30 | Chitwan | *T. asperelloides* | Green | Inner terai | 212 | 15.7 | 29.2 | 1993 |
| N31 | Banke | *T. asperelloides* | Blue | Terai | 151 | 15.4 | 31.6 | 1175 |
| NT32 | Banke | *T. asperelloides* | Orange | Terai | 151 | 15.4 | 31.6 | 1175 |
| NT33 | Banke | *T. asperelloides* | Green | Terai | 151 | 15.4 | 31.6 | 1175 |
| NT34 | Banke | *T. asperelloides* | Green | Terai | 151 | 15.4 | 31.6 | 1175 |
| NT35 | Banke | *T. asperelloides* | Orange | Terai | 151 | 15.4 | 31.6 | 1175 |
| NT36 | Banke | *T. asperelloides* | Orange | Terai | 151 | 15.4 | 31.6 | 1175 |
| NT37 | Banke | *T. asperelloides* | Green | Terai | 151 | 15.4 | 31.6 | 1175 |
| NT38 | Banke | *T. asperelloides* | Orange | Terai | 151 | 15.4 | 31.6 | 1175 |
| NT39 | Banke | *T. asperelloides* | Orange | Terai | 151 | 15.4 | 31.6 | 1175 |
| OT40 | Ohio | *T. hamatum* | Sky blue | Ohio | 97 | 3.2 | 27.0 | 1257 |
| OT41 | Ohio | *T. ghanense* | Pink | Ohio | 97 | 3.2 | 27.0 | 1257 |
| NT42 | Banke | *T. asperelloides* | Green | Terai | 151 | 15.4 | 31.6 | 1175 |
| NT43 | Banke | *T. asperelloides* | Green | Terai | 151 | 15.4 | 31.6 | 1175 |
| ^x^ Collection, molecular characterization and species identification of *Trichoderma* isolates (Khadka and Miller, unpublished).  ^xy^ NA = Not applicable  ^xy^ Terai = Lowland region (67- 300 m)  ^z^ Source for climate data: Climate data org (<https://en.climate-data.org>). | | | | | | | | |

**Supplementary Table S2.**Mean and rank performances of *Trichoderma* isolates cultured *in-vitro* under three levels of osmotic stress.

|  | Matric water potential  -2.8 Ψ | |  | Matric water potential  -4.8 Ψ | |  | Matric water potential  -8.5 Ψ | |  |  |
| --- | --- | --- | --- | --- | --- | --- | --- | --- | --- | --- |
| Isolate name | Radial mycelial growth (cm)^x^ | Rank^y^ |  | Radial mycelial growth (cm)^x^ | Rank^y^ |  | Radial mycelial growth (cm)^x^ | Rank^y^ |  | Average Rank^z^ |
| NT1 | 5.62 ± 1.88 | 28.0 |  | 3.58 ± 2.18 | 23.0 |  | 0.85 ± 0.61 | 30.0 |  | 27.0 |
| NT2 | 7.83 ± 1.67 | 10.0 |  | 3.80 ± 1.67 | 20.0 |  | 1.22 ± 0.38 | 5.5 |  | 11.8 |
| NT3 | 9.03 ± 0.97 | 6.0 |  | 4.51 ± 1.24 | 8.0 |  | 1.23 ± 0.23 | 4.0 |  | 6.0 |
| NT4 | 4.57 ± 2.62 | 38.0 |  | 2.78 ± 1.00 | 36.0 |  | 0.59 ± 0.09 | 42.0 |  | 38.7 |
| NT5 | 4.97 ± 1.47 | 31.0 |  | 1.15 ± 0.65 | 44.0 |  | 0.48 ± 0.02 | 44.0 |  | 39.7 |
| NT6 | 7.07 ± 0.17 | 16.0 |  | 3.10 ± 1.13 | 32.0 |  | 0.86 ± 0.18 | 28.5 |  | 25.5 |
| NT7 | 4.94 ± 2.73 | 33.0 |  | 3.36 ± 1.39 | 28.0 |  | 0.98 ± 0.48 | 17.5 |  | 26.2 |
| NT8 | 10.00 ± 0.00 | 1.5 |  | 5.12 ± 1.90 | 3.0 |  | 0.80 ± 0.03 | 35.0 |  | 13.2 |
| NT9 | 6.93 ± 1.58 | 18.0 |  | 3.57 ± 1.40 | 24.0 |  | 0.91 ± 0.39 | 24.0 |  | 22.0 |
| NT10 | 4.70 ± 2.25 | 36.0 |  | 3.30 ± 1.43 | 30.0 |  | 0.77 ± 0.23 | 37.0 |  | 34.3 |
| NT11 | 4.89 ± 3.44 | 34.0 |  | 2.35 ± 1.00 | 41.0 |  | 0.96 ± 0.24 | 20.0 |  | 31.7 |
| NT12 | 6.63 ± 2.70 | 22.0 |  | 4.42 ± 1.15 | 12.0 |  | 1.00 ± 0.33 | 15.5 |  | 16.5 |
| NT13 | 3.70 ± 1.22 | 43.0 |  | 1.64 ± 0.54 | 42.0 |  | 0.61 ± 0.09 | 40.5 |  | 41.8 |
| TN14 | 7.25 ± 1.25 | 14.0 |  | 4.37 ± 2.48 | 13.0 |  | 0.95 ± 0.43 | 21.0 |  | 16.0 |
| NT15 | 4.43 ± 3.06 | 40.0 |  | 2.88 ± 1.66 | 34.0 |  | 0.82 ± 0.28 | 32.5 |  | 35.5 |
| NT16 | 6.87 ± 1.30 | 20.0 |  | 4.46 ± 1.78 | 10.0 |  | 1.19 ± 0.44 | 7.0 |  | 12.3 |
| NT17 | 5.72 ± 4.28 | 27.0 |  | 2.75 ± 2.20 | 37.0 |  | 1.11 ± 0.53 | 10.5 |  | 24.8 |
| NT18 | 7.18 ± 0.52 | 15.0 |  | 3.47 ± 1.28 | 25.0 |  | 0.93 ± 0.33 | 22.0 |  | 20.7 |
| NT19 | 3.63 ± 2.68 | 44.0 |  | 1.36 ± 0.33 | 43.0 |  | 0.57 ± 0.07 | 43.0 |  | 43.3 |
| NT20 | 4.63 ± 2.71 | 37.0 |  | 3.31 ± 1.94 | 29.0 |  | 1.00 ± 0.13 | 15.5 |  | 27.2 |
| NT21 | 6.90 ± 1.03 | 19.0 |  | 3.86 ± 1.79 | 19.0 |  | 0.88 ± 0.34 | 26.0 |  | 21.3 |
| NT22 | 10.00± 0.00 | 1.5 |  | 5.10 ± 1.98 | 4.0 |  | 1.18 ± 0.00 | 8.0 |  | 4.5 |
| NT23 | 4.11± 2.56 | 41.0 |  | 2.92 ± 1.38 | 33.0 |  | 0.73 ± 0.21 | 38.0 |  | 37.3 |
| NT24 | 5.85 ± 1.15 | 26.0 |  | 2.70 ± 1.08 | 39.0 |  | 0.88 ± 0.34 | 26.0 |  | 30.3 |
| NT25 | 4.95 ± 0.90 | 32.0 |  | 3.75 ± 1.65 | 21.0 |  | 0.86 ± 0.33 | 28.5 |  | 27.2 |
| NT26 | 5.21 ± 2.46 | 30.0 |  | 4.23 ± 2.13 | 14.5 |  | 0.83 ± 0.14 | 31.0 |  | 25.2 |
| NT27 | 3.85 ± 2.32 | 42.0 |  | 2.38 ±1.48 | 40.0 |  | 0.82 ± 0.28 | 32.5 |  | 38.2 |
| NT28 | 6.17 ± 1.50 | 25.0 |  | 3.38 ± 0.97 | 27.0 |  | 0.88 ± 0.29 | 26.0 |  | 26.0 |
| NT29 | 8.07 ± 0.17 | 9.0 |  | 4.48 ± 2.00 | 9.0 |  | 1.13 ± 0.44 | 9.0 |  | 9.0 |
| NT30 | 5.40 ± 2.77 | 29.0 |  | 4.85 ± 3.08 | 5.0 |  | 1.04 ± 0.13 | 13.0 |  | 15.7 |
| N31 | 9.17 ± 0.50 | 3.5 |  | 5.44 ± 2.08 | 1.0 |  | 1.03 ± 0.31 | 14.0 |  | 6.2 |
| NT32 | 7.06 ± 2.94 | 17.0 |  | 3.18 ± 1.78 | 31.0 |  | 1.05 ± 0.35 | 12.0 |  | 20.0 |
| NT33 | 9.09 ± 0.91 | 5.0 |  | 4.43 ± 1.64 | 11.0 |  | 1.11 ± 0.58 | 10.5 |  | 8.8 |
| NT34 | 9.17 ± 0.50 | 3.5 |  | 3.89 ± 0.94 | 18.0 |  | 1.24 ± 0.39 | 3.0 |  | 8.2 |
| NT35 | 6.59 ± 3.14 | 23.0 |  | 5.15 ± 2.45 | 2.0 |  | 1.22 ± 0.58 | 5.5 |  | 10.2 |
| NT36 | 7.63 ± 2.20 | 12.0 |  | 4.23 ± 2.10 | 14.5 |  | 0.97± 0.27 | 19.0 |  | 15.2 |
| NT37 | 7.69 ± 0.64 | 11.0 |  | 4.64 ± 2.88 | 7.0 |  | 1.43 ± 0.54 | 1.0 |  | 6.3 |
| NT38 | 6.37 ± 3.63 | 24.0 |  | 4.13 ± 1.46 | 16.0 |  | 0.98 ± 0.35 | 17.5 |  | 19.2 |
| NT39 | 6.82 ± 2.02 | 21.0 |  | 4.09 ± 2.78 | 17.0 |  | 0.61 ± 0.01 | 40.5 |  | 26.3 |
| OT40 | 4.48 ± 3.10 | 39.0 |  | 2.83 ± 1.43 | 35.0 |  | 0.68 ± 0.05 | 39.0 |  | 37.7 |
| OT41 | 4.73 ± 3.00 | 35.0 |  | 2.72 ± 1.38 | 38.0 |  | 0.80 ± 0.13 | 36.0 |  | 36.3 |
| NT42 | 8.66 ± 0.18 | 7.0 |  | 3.42 ± 2.72 | 26.0 |  | 0.80 ± 0.25 | 34.0 |  | 22.3 |
| NT43 | 8.44 ± 1.56 | 8.0 |  | 3.73 ± 0.65 | 22.0 |  | 0.92 ± 0.15 | 23.0 |  | 17.7 |
| T22 | 7.58 ± 0.42 | 13.0 |  | 4.83 ± 2.31 | 6.0 |  | 1.28 ± 0.61 | 2.0 |  | 7.0 |

^x^ Values represent the mean radial growth ± SE of isolates grown on three different matric water potential (-2.8 Ψ, -4.8Ψ, -8.5Ψ) from two trials each performed in triplicate.

^y^ Ranking of *Trichoderma* isolates based on their mycelial growth in PEG amended PDA media.

^z^ Average rank of *Trichoderma* isolates was calculated by averaging the growth of *Trichoderma* isolates in three different matric water potential (-2.8 Ψ, -4.8Ψ, -8.5Ψ).

**Supplementary Table S3.** Test of main effects, interaction, and slices of interaction based on the fit of a linear mixed model (ANOVA) for 13 *Trichoderma* isolates and irrigation on shoot and root biomass of ‘Roma Organic’ tomato.

| **Source of Variation** | | **Num DF** | **Den DF** | **F value** | **Pr> F** |
| --- | --- | --- | --- | --- | --- |
| **Shoot Biomass** | | | | | |
| *Trichoderma* isolates (T) | | 12 | 178 | 2.47 | 0.005 |
| Irrigation (I) | | 1 | 178 | 511.85 | <0.0001 |
| T X I | | 12 | 178 | 1.28 | 0.2 |
| Slices | |  |  |  |  |
|  | T – Water deficit stress | 12 | 88 | 2.19 | 0.005 |
|  | T – Irrigated | 12 | 88 | 1.49 | 0.1 |
| **Root Biomass** | | | | | |
| *Trichoderma* isolates (T) | | 12 | 168 | 0.95 | 0.5 |
| Irrigation (I) | | 1 | 168 | 93.30 | <0.0001 |
| T X I | | 12 | 168 | 1.31 | 0.2 |
|  | T – Water deficit stress | 12 | 88 | 3.06 | 0.01 |
|  | T – Irrigated | 12 | 88 | 0.65 | 0.8 |

**Supplementary Table S4.**  Test of main effects, interaction, and slices of interaction based on the fit of a linear mixed model (ANOVA) for four *Trichoderma* isolates on shoot and root biomass of three tomato genotypes, ‘Jaune Flamme’, ‘Roma Organic’, and ‘Punta Banda’ under water deficit stress and irrigated conditions.

| **Source of Variation** | | **Num DF** | **Den DF** | **F value** | **Pr> F** |
| --- | --- | --- | --- | --- | --- |
| **Shoot Biomass** | | | | | |
| *Trichoderma* isolates (T) | | 4 | 477 | 38.74 | <0.0001 |
| Irrigation (I) | | 1 | 477 | 876.64 | <0.0001 |
| Genotypes (G) | | 2 | 477 | 16.38 | <0.0001 |
| T X I | | 4 | 477 | 1.34 | 0.25 |
| T X G | | 8 | 477 | 1.86 | 0.065 |
| G X I | | 2 | 477 | 9.97 | <0.0001 |
| T X I X G | | 8 | 477 | 1.52 | 0.14 |
| Slices | |  |  |  |  |
|  | T X G – Water deficit stress | 14 | 477 | 10.81 | <0.0001 |
|  | T X G – Irrigated | 14 | 477 | 6.24 | <0.0001 |
| **Root Biomass** | | | | | |
| *Trichoderma* isolates (T) | | 4 | 478 | 8.29 | <0.0001 |
| Irrigation (I) | | 1 | 478 | 120.63 | <0.0001 |
| Genotypes (G) | | 2 | 478 | 289.70 | <0.0001 |
| T X I | | 4 | 478 | 1.32 | 0.3 |
| T X G | | 8 | 478 | 1.52 | 0.14 |
| G X I | | 2 | 478 | 15.46 | <0.0001 |
| T X I X G | | 8 | 478 | 1.33 | 0.2 |
| Slices | |  |  |  |  |
|  | T X G – Water deficit stress | 14 | 478 | 16.03 | <0.0001 |
|  | T X G – Irrigated | 14 | 478 | 31.75 | <0.0001 |

**Supplementary Table S5.**  Test of main effects, interaction, and slices of interaction based on the fit of a linear mixed model (ANOVA) for four *Trichoderma* isolates on the number, chlorophyll content, relative water content of leaves on three tomato genotypes, ‘Jaune Flamme’, ‘Roma Organic’, and ‘Punta Banda’ under water deficit stress and irrigated conditions.

| **Source of Variation** | | **Num DF** | **Den DF** | **F value** | **Pr > F** |
| --- | --- | --- | --- | --- | --- |
| **Leaf Number** | | | | | |
| *Trichoderma* isolates (T) | | 4 | 369 | 6.09 | <0.0001 |
| Irrigation (I) | | 1 | 369 | 538.42 | <0.0001 |
| Genotypes (G) | | 2 | 369 | 71.18 | <0.0001 |
| T X I | | 4 | 369 | 2.99 | 0.01 |
| T X G | | 8 | 369 | 1.15 | 0.3 |
| G X I | | 2 | 369 | 6.60 | 0.002 |
| T X I X G | | 8 | 369 | 2.26 | 0.02 |
| Slices | |  |  |  |  |
|  | T X G – Water deficit stress | 14 | 369 | 5.66 | <0.0001 |
|  | T X G – Irrigated | 14 | 369 | 10.30 | <0.0001 |
| **Leaf Chlorophyll Content** | | | | | |
| *Trichoderma* isolates (T) | | 4 | 370 | 17.52 | <0.0001 |
| Irrigation (I) | | 1 | 370 | 8.70 | 0.003 |
| Genotypes (G) | | 2 | 370 | 95.47 | <0.0001 |
| T X I | | 4 | 370 | 2.24 | 0.06 |
| T X G | | 8 | 370 | 2.88 | 0.004 |
| G X I | | 2 | 370 | 9.63 | <0.0001 |
| T X I X G | | 8 | 370 | 1.23 | 0.3 |
| Slices | |  |  |  |  |
|  | T X G – Water deficit stress | 14 | 370 | 9.04 | <0.0001 |
|  | T X G – Irrigated | 14 | 370 | 14.09 | <0.0001 |
| **Relative Water Content** | | | | | |
| *Trichoderma* isolates (T) | | 4 | 102 | 3.57 | 0.009 |
| Irrigation (I) | | 1 | 102 | 222.9 | <0.0001 |
| Genotypes (G) | | 2 | 102 | 7.72 | 0.0007 |
| T X I | | 4 | 102 | 3.56 | 0.009 |
| T X G | | 8 | 102 | 2.66 | 0.03 |
| G X I | | 2 | 102 | 7.34 | 0.001 |
| T X I X G | | 8 | 102 | 1.67 | 0.1 |
| Slices | |  |  |  |  |
|  | T X G – Water deficit stress | 14 | 102 | 2.09 | 0.05 |
|  | T X G – Irrigated | 14 | 102 | 0.27 | 1.0 |

**Supplementary Table S6**. Test of main effects, interaction, and slices of interaction based on the fit of a linear mixed model (ANOVA) for four *Trichoderma* isolates on the stomatal conductance, net photosynthesis, and quantum efficiency of leaves of three tomato genotypes, ‘Jaune Flamme’, ‘Roma Organic’, and ‘Punta Banda’ under water deficit stress and irrigated conditions.

| **Source of Variation** | | **Num DF** | **Den DF** | **F value** | **Pr> F** |
| --- | --- | --- | --- | --- | --- |
| **Stomatal Conductance** | | | | | |
| *Trichoderma* isolates (T) | | 4 | 73 | 2.19 | 0.08 |
| Irrigation (I) | | 1 | 73 | 462.50 | <0.0001 |
| Genotypes (G) | | 2 | 73 | 2.22 | 0.1 |
| T X I | | 4 | 73 | 2.27 | 0.07 |
| T X G | | 8 | 73 | 0.61 | 0.8 |
| G X I | | 2 | 73 | 2.37 | 0.07 |
| T X I X G | | 8 | 73 | 0.59 | 0.8 |
| Slices | |  |  |  |  |
|  | T X G – Water deficit stress | 14 | 73 | 2.54 | 0.03 |
|  | T X G – Irrigated | 14 | 73 | 0.60 | 0.8 |
| **Net Photosynthesis** | | | | | |
| *Trichoderma* isolates (T) | | 4 | 73 | 2.26 | 0.07 |
| Irrigation (I) | | 1 | 73 | 617.34 | <0.0001 |
| Genotypes (G) | | 2 | 73 | 32.99 | <0.0001 |
| T X I | | 4 | 73 | 1.24 | 0.3 |
| T X G | | 8 | 73 | 2.09 | 0.05 |
| G X I | | 2 | 73 | 44.31 | <0.0001 |
| T X I X G | | 8 | 73 | 1.29 | 0.3 |
| Slices | |  |  |  |  |
|  | T X G – Water deficit stress | 14 | 73 | 2.54 | 0.07 |
|  | T X G – Irrigated | 14 | 73 | 10.14 | 1.0 |
